# Blood–brain barrier penetrating neprilysin degrades monomeric amyloid-beta in a mouse model of Alzheimer’s disease

**DOI:** 10.1186/s13195-022-01132-2

**Published:** 2022-12-05

**Authors:** Fadi Rofo, Nicole G. Metzendorf, Cristina Saubi, Laura Suominen, Ana Godec, Dag Sehlin, Stina Syvänen, Greta Hultqvist

**Affiliations:** 1 Department of Pharmacy, Uppsala University, Biomedicinskt Centrum BMC, Husargatan 3, 751 24 Uppsala, Sweden; 2 Department of Public Health and Caring Sciences, Uppsala University, Uppsala, Sweden

**Keywords:** Amyloid-β (Aβ), Neprilysin (NEP), Blood–brain barrier (BBB), Transferrin receptor (TfR), Recombinant proteins

## Abstract

**Background:**

Aggregation of the amyloid-β (Aβ) peptide in the brain is one of the key pathological events in Alzheimer’s disease (AD). Reducing Aβ levels in the brain by enhancing its degradation is one possible strategy to develop new therapies for AD. Neprilysin (NEP) is a membrane-bound metallopeptidase and one of the major Aβ-degrading enzymes. The secreted soluble form of NEP (sNEP) has been previously suggested as a potential protein-therapy degrading Aβ in AD. However, similar to other large molecules, peripherally administered sNEP is unable to reach the brain due to the presence of the blood–brain barrier (BBB).

**Methods:**

To provide transcytosis across the BBB, we recombinantly fused the TfR binding moiety (scFv8D3) to either sNEP or a previously described variant of NEP (muNEP) suggested to have higher degradation efficiency of Aβ compared to other NEP substrates, but not per se to degrade Aβ more efficiently. To provide long blood half-life, an Fc-based antibody fragment (scFc) was added to the designs, forming sNEP-scFc-scFv8D3 and muNEP-scFc-scFv8D3. The ability of the mentioned recombinant proteins to degrade Aβ was first evaluated in vitro using synthetic Aβ peptides followed by sandwich ELISA. For the in vivo studies, a single injection of 125-iodine-labelled sNEP-scFc-scFv8D3 and muNEP-scFc-scFv8D3 was intravenously administered to a tg-ArcSwe mouse model of AD, using scFc-scFv8D3 protein that lacks NEP as a negative control. Different ELISA setups were applied to quantify Aβ concentration of different conformations, both in brain tissues and blood samples.

**Results:**

When tested in vitro, sNEP-scFc-scFv8D3 retained sNEP enzymatic activity in degrading Aβ and both constructs efficiently degraded arctic Aβ*.* When intravenously injected, sNEP-scFc-scFv8D3 demonstrated 20 times higher brain uptake compared to sNEP. Both scFv8D3-fused NEP proteins significantly reduced aggregated Aβ levels in the blood of tg-ArcSwe mice, a transgenic mouse model of AD, following a single intravenous injection. In the brain, monomeric and oligomeric Aβ were significantly reduced. Both scFv8D3-fused NEP proteins displayed a fast clearance from the brain.

**Conclusion:**

A one-time injection of a BBB-penetrating NEP shows the potential to reduce, the likely most toxic, Aβ oligomers in the brain in addition to monomers. Also, Aβ aggregates in the blood were reduced.

**Supplementary Information:**

The online version contains supplementary material available at 10.1186/s13195-022-01132-2.

## Introduction

Alzheimer’s disease (AD) is a progressive neurodegenerative disorder and the most common form of dementia. It is characterized by the extracellular deposition of amyloid plaques and the formation of intracellular neurofibrillary tangles comprised of tau protein [1]. Amyloid plaques are mainly comprised of amyloid-beta (Aβ) peptides. Aβ is formed by enzymatic digestion of Aβ precursor protein (APP), giving rise to Aβ peptides of different lengths [2]. The 40 amino acid long peptide (Aβ40) is the most abundant Aβ form in the human brain followed by Aβ42, an isoform that is more prone to aggregate. Under physiological condition, Aβ exists as an unfolded monomer with studies demonstrating possible physiological roles of this peptide in neurogenesis and lipid homeostasis [3–6]. However, during AD, partially folded Aβ monomers start to form aggregates of different sizes and solubility, ranging from soluble oligomers with hairpins and protofibrils to insoluble fibrils and finally amyloid plaques [7, 8]. Soluble aggregates (oligomers and protofibrils) have been demonstrated to correlate with AD progression and are suggested to be the most neurotoxic species of Aβ [9–13]. Accumulation of Aβ in the brain during AD is likely to be a result of an imbalance between its formation, aggregation and clearance [14]. The majority of anti-AD treatments, mainly antibody therapies, have focused on reducing the formation of larger aggregates of Aβ [15]. Nevertheless, experimental treatments aiming at enhancing the degradation of Aβ have also been discussed. These studies have focused on identifying and enhancing the activity of several endopeptidases present in the brain that are capable of degrading Aβ [16–18].

One of the major enzymes capable of degrading Aβ into inactive fragments is neprilysin (NEP) [19]. NEP was first discovered in the kidneys as a 749 amino acid long zinc metallopeptidase [20, 21]. The enzyme is also expressed in several other tissues such as the brain, lungs, intestines, heart and the brain. In the brain, NEP is highly expressed in the hippocampus [17] and in areas associated with AD pathology [22] and the levels are lower in patients with AD [23, 24]. NEP is a transmembrane protein comprising a large extracellular domain containing the catalytic site, a transmembrane domain and a small cytoplasmic domain [20]. NEP can be released from the cell membrane, giving rise to soluble NEP (sNEP). sNEP is a non-membrane-bound form of NEP with retained catalytic activity and biodistribution in several tissues and biological fluids [25].

The first evidence of NEP’s role in regulating Aβ levels comes from an in vitro study demonstrating the degradation of Aβ but not its precursor APP using recombinant NEP [26]. Since then, several studies have highlighted the significant role of NEP in AD pathology [27–29]. Importantly, accumulation of Aβ and cognitive impairment have been observed in NEP knock-out mice [30] and following treatment of preclinical animal models of AD with NEP antagonists [31, 32]. Furthermore, NEP gene therapy and transgenic overexpression of NEP have been associated with a significant reduction in the concentration of Aβ in the brain [33, 34]. We have recently displayed the therapeutic significance of enhancing NEP activity in the brain using a brain-targeting somatostatin peptide, resulting in a significant increase in the degradation of Aβ42 in the hippocampus of AD mice [17]. All these studies have highlighted the potential of NEP as a target for AD therapy.

Despite its therapeutic significance in degrading Aβ, there are still some challenges that impede the use of NEP as a protein therapy in AD. Similar to other large molecules, NEP is unable to pass the blood–brain barrier (BBB) and reach intra-brain targets. All previous studies that have demonstrated the protective role of recombinant NEP protein were either performed in vitro [35] or used intra-cerebral administration of NEP to by-pass the BBB resulting in NEP distribution to a limited part of the brain [36]. To our knowledge, the only study using peripherally administered recombinant NEP protein with enhanced BBB transport was performed in wild-type (WT) rats without the inclusion of animal models of AD pathology [16]. In the current study, we sought to use the previously described BBB transporter (scFv8D3) that is derived from the rat anti-mouse transferrin receptor (TfR) antibody 8D3 and has shown to significantly improve brain uptake of protein drugs [17, 37, 38]. When attached to a therapeutic protein, it has also enabled therapeutic effect in the brain parenchyma [17, 17, 39–41] and when attached to proteins labelled with 124-iodine [^124^I], it has enabled the detection of Aβ aggregates in the mouse brain [38, 42]. To produce the brain-penetrating recombinant protein sNEP-scFc-scFv8D3, the brain transporter scFv8D3 moiety was first linked to a single-chain fragment constant (scFc) of mouse IgG2c antibody to prolong the half-life of the whole construct, which in turn was further fused to sNEP.

Another challenge in using NEP as an anti-AD therapy is attributed to the substrate specificity of the enzyme. In addition to Aβ, NEP is capable of degrading other peptides involved in several physiological processes such as blood pressure regulation and nociception. Examples include neuropeptide Y, substance P, angiotensin I and II, bradykinin and neurotensin [43, 44]. Therefore, systematic administration of NEP may lead to degradation of other physiologically relevant peptides causing unwanted adverse effects. Another group has previously shown that a mutated variant of NEP (muNEP) with two mutations (G399V/G714K), resulted in their hands in an up to 3200-fold reduction in the capacity to degrade other NEP substrates [44]. The capacity the muNEP had in degrading Aβ40 compared to the wild-type NEP varied depending on a Aβ concentration with what seemed to be substantially reduced activity at low concentrations and a 20-fold enhanced ability to degrade at very high concentrations. For this reason, we also engineered a second protein (muNEP-scFc-scFv8D3) [44].

The aim of the current study was to evaluate Aβ degradation capacity of the BBB-penetrant soluble NEP protein (sNEP-scFc-scFv8D3) and the BBB-penetrant mutated variant of NEP (muNEP-scFc-scFv8D3), both in vitro and in vivo using a transgenic mouse model of AD. The mouse model with AD-like Aβ pathology (tg-ArcSwe) was used in this study. This model harbours both the Arctic and Swedish mutations in the APP gene (APP E693G (or Aβ E22G) and APP KM670/671NL) [45]. The Arctic mutation, which is located in the middle of Aβ, facilitates the formation of Aβ aggregates, but preferably smaller protofibrils [46]. The Swedish mutation increases Aβ production. Amino acid 22 in Aβ, which is mutated in the arctic, has been predicted to have a very strong interaction with NEP [47]. Importantly, these mice have arctic-Aβ (from the transgene) that is mainly present as aggregates in the brain and mouse-Aβ that is present mostly as monomers which will be the predominant species in blood. In the current study, we aimed to study the degradation efficiency of BBB-penetrant NEP proteins on the different forms of Aβ present in tg-ArcSwe mice.

## Materials and methods

### Protein cloning, expression and purification

The protein constructs were cloned into pcDNA3.4 vector (GeneArt, Regensburg, Germany), amplified in *Escherichia coli* and recombinantly expressed using Expi293 cells as described previously [48]. Seven days after transfection, the proteins were purified using the ÄKTA start system and protein G columns (Cytiva, Uppsala, Sweden) as described previously [49]. The eluted proteins were aliquoted and stored at − 80 °C until further application.

### SDS-PAGE and Tycho nt.6

The eluted proteins were mixed with 25% LDS sample buffer (Thermo Scientific, Waltham, MA) and loaded onto 4–12% Bis–Tris protein gels (Thermo Scientific, Waltham, MA). The gel was then stained with PAGE blue protein solution (Thermo Scientific, Waltham, MA) as described previously [49]. Furthermore, to evaluate the stability of the purified proteins, Tycho nt.6 instrument (NanoTemper technologies, München, Germany) was used as previously described [49]. Briefly, 8 μL of the purified proteins (final concentration 1 μM) was heated in a glass capillary with linearly increasing temperature and fluorescence intensities at 330 nm and 350 nm were recorded.

### TfR and Aβ binding ELISA

For TfR ELISA, 96-well half-area plates (Corning Inc., Corning, NY) were coated overnight at 4 °C with 500 ng/mL of mouse TfR protein (recombinantly prepared in the lab). This was followed by 2-h incubation with ELISA blocking buffer (1% BSA in PBS) at RT.

For Aβ ELISA, 96-well half-area plates (Corning Inc., Corning, NY) were coated overnight at 4 °C with 1 μg/mL of rabbit polyclonal antibodies detecting the C-terminal of Aβ (44–136, Thermo Scientific, Waltham, MA) (our in-house experiments show that it detects both Aβ40 and 42) or Aβ1-42 (700,254, Thermo Scientific, Waltham, MA). This was followed by 2-h incubation with ELISA blocking buffer (1% BSA in PBS) at RT, followed by additional 2-h incubation with 1 nM of Aβ1-40 monomers (Innovagen, Lund, Sweden) or 1 nM of Aβ1-42 aggregates, prepared as previously described [50]. For both ELISAs, serial dilutions of the recombinant proteins were prepared in ELISA incubation buffer (0.1% BSA, 0.05% Tween-20 in PBS) and added to the plates. The plates were incubated for 2 h at RT, followed by the addition of a goat anti-mouse HRP secondary antibody (Sigma Aldrich, Stockholm, Sweden) for 1 h at RT. K-blue aqueous TMB (Neogen Corp, Lexington, KY) was used for signal development and 1 M H_2_SO_4_ as the stop solution. Using a Spark multimode microplate reader (Tecan, Männedorf, Switzerland), absorbance was measured at 450 nm.

### Aβ degradation assay in vitro

The ability of sNEP-scFc-scFv8D3 and muNEP-scFc-scFv8D3 to degrade Aβ in vitro was evaluated using an assay similar to what has been described previously with some modifications [35]. sNEP (NovusBio, Centennial, CO) was used as the positive control and PBS as the negative control. Synthetic Aβ1-40, Aβ1-42 or arctic Aβ1-42 (final concentration 0.5 μM or 2.5 μM) (Innovagen, Lund, Sweden; Sigma Aldrich, Stockholm, Sweden) were mixed with a fixed concentration of 0.5 μM of the recombinant proteins. Recombinant NEP proteins were added immediately to the Aβ peptides and mixtures and incubated at 37 °C for 2.5, 24, 48 and 72 h. The reaction was stopped by immediately freezing the mixtures at − 20 °C. A mixture of Aβ and PBS at 0 h was used as the baseline. Degradation of Aβ in the different mixtures was assessed using a sandwich ELISA setup similar to what has been previously described [17]. In this ELISA, a capture antibody that detected the C-terminal end of Aβ and a detection antibody that detected the N-terminal of Aβ were used. Therefore, such a setup detected full-length Aβ only (but both monomers and aggregates) and excluded the Aβ fragments produced by NEP degradation. In this ELISA, 96-well half-area plates (Corning Inc., Corning, NY) were coated overnight at 4 °C with rabbit polyclonal antibodies detecting the C-terminal of Aβ (44–136, Thermo Scientific, Waltham, MA) or Aβ 1–42 (700,254, Thermo Scientific, Waltham, MA). Following blocking with 1% BSA in PBS, mixtures of Aβ/NEP proteins were diluted in ELISA incubation buffer (0.1% BSA, 0.05% Tween-20 in PBS) and added to the plates for 2 h at RT. Biotinylated 3D6 antibody (recombinantly produced in the lab) was added to the plates and incubated for 1 h at RT. This was followed by the addition of Streptavidin-HRP (Mabtech, Stockholm, Sweden) and the plates were further incubated for 1 h at RT. Signals were developed as described above.

### Radiochemistry

Equimolar amounts of sNEP-scFc-scFv8D3 (corresponding to 40 μg, Mw 162 kDa), muNEP-scFc-scFv8D3 (corresponding to 40 μg, Mw 162 kDa) and scFc-scFv8D3 (corresponding to 20 μg, Mw 81 kDa) were labelled with 9.5 MBq of 125-iodine (^125^I) (Perkin Elmer Inc., Waltham, USA) as described previously [51]. This reaction will add ^125^I randomly on all accessible tyrosines on the protein. The reaction was initiated by the addition of 5 μg of Chloramine-T (Sigma Aldrich, Stockholm, Sweden) for 90 s and stopped by the addition of 10 μg of sodium metabisulphite (Sigma Aldrich, Stockholm, Sweden). Free and unbound iodine is washed away from the labelled protein using Zeba desalting columns with a 7-kDa molecular weight cutoff (Cat. No. 89892, Thermofisher). The labelling yield was around 60% for the trace dose experiment and around 80% and the resulting specific activity was 28 MBq/nmol for the three proteins for the therapeutic study. For therapeutic studies, 10% of labelled proteins were mixed with 90% unlabelled proteins. The quality of the ^125^I-labelled proteins was analysed by instant thin layer chromatography (iTLC) by applying 1 µL of the labelled proteins onto a silicia-coated alumina plate (TLC plate, Merck, Darmstadt, Germany). Samples were separated with 70% (v/v) acetone as a solvent. The TLC plate was developed with an X-ray film for approximately 1 min and the film was measured by using a Cyclone Phosphoimager (Perkin Elmer, Waltham, USA). Obtained images were analysed with ImageJ [52].

### Animals

The tg-ArcSwe mouse model was used in this study. This is a transgenic mouse model of AD-like Aβ pathology harbouring both the Arctic and Swedish mutations in the APP gene (APP E693G and APP KM670/671NL) [45]. Since previous studies have demonstrated a higher proteolytic efficiency of NEP on Aβ monomers compared to aggregates [33, 53], Tg-ArcSwe mice of 7–8 months old were used. At this age, these mice have a higher proportion of soluble Aβ aggregates compared to insoluble plaques than in older mice [54]. Both females and males were included (weight range: females 21.2–30 g, males 30.8–36 g). The mice were housed under daily surveillance of well-trained personnel in individually ventilated cages with 4–5 mice per cage in an animal house at Uppsala University. The animals had free access to food and water, a 12:12-h light:dark cycle, a humidity of 50–55% and a constant temperature of 22–23 °C. All procedures were carried out according to the Swedish ethical policies regarding animal experiments and following the ARRIVE guidelines. The ethical permit was approved by the Uppsala County Animal Ethics Board (#5.8.18–13,350/17 and #5.8.18–20,401/2020) and carried out according to the regulations of the Swedish Animal Welfare Agency and complied with the European Communities Council Directive of 22 September 2022 (2010/63/E.U.).

### Therapeutic study and ex vivo analysis

For the therapeutic experiments using both tracer and therapeutic doses, the mice were injected intravenously (bolus) with ^125^I-labelled proteins in the tail vein. In the tracer dose study, 0.05 mg/kg body weight (0.3 nmol/kg body weight) of ^125^I-labelled sNEP as well as sNEP-scFc-scFv8D3 (100% ^125^I labelled) was injected. The animals were euthanized after 2 h (sNEP *n* = 5, sNEP-scFc-scFv8D3 *n* = 3), 6 h (sNEP-scFc-scFv8D3 *n* = 3) and 72 h (sNEP-scFc-scFv8D3 *n* = 3) by perfusion with 0.9% NaCl under terminal isoflurane anaesthesia. In the therapeutic study, mice were injected ^125^I-labelled proteins with a dose of 5 mg/kg body weight of [^125^I]sNEP-scFc-scFv8D3 or [^125^I]muNEP-scFc-scFv8D3 (corresponding to around 30 nmol/kg body weight; Mw 162 kDa, sNEP-scFc-scFv8D3 *n* = 4, muNEP-scFc-scFv8D3 *n* = 3) or 2.5 mg/kg of [^125^I]scFv8D3-scFc (corresponding to around 30 nmol/kg body weight; Mw 81 kDa, *n* = 4) but only 10% of the total injected protein concentration were labelled with ^125^I. Blood samples were collected after 1, 4, 6, 24, 48 and 72 h to determine the blood half-life of the injected proteins. The mice were euthanized after 72 h by transcardic perfusion with 0.9% NaCl under terminal isoflurane anaesthesia. In both studies, the brain, blood and peripheral organs were collected after the perfusion. The brain was divided into two hemispheres, and the cerebellum was separated from the left hemisphere. The concentration of the radiolabelled proteins was determined by measuring the radioactivity in blood samples, brain parts and organs by using a Wizard 2470 gamma counter (Perkin Elmer Inc., Waltham, USA) as described previously [17, 41]. Based on the measured radioactivity in the tissues and blood (counts per minute, CPM), the concentration of the radioactivity was quantified as percentage of injected dose (%ID) per gramme tissue or blood.

Blood was separated into blood pellet and plasma by centrifugation 10, 000 × g, 5 min at 4 °C. Two microlitres of plasma as well as urine was added onto a TLC plate (Merck, Darmstadt, Germany), airdried and separated with 70% (v/v) acetone iTLC. The TLC plate was developed with an X-ray film and red in a Cyclone Phosphoimager (Perkin Elmer Inc., Waltham, USA). Images were analysed with ImageJ [52].

The right hemisphere was used for immunohistochemistry analysis, while the left hemisphere was homogenized to obtain different extracts of Aβ. First, tris-buffered saline (TBS) containing cOmplete™ protease inhibitors (Roche, Basel, Switzerland) was added to the left hemisphere and the mix was homogenized using a Precellys Evolution System (Bertin Corp, Rockville, MD). The homogenates were subsequently centrifuged for 1 h at 16,000 × g at 4 °C to generate a brain extract of soluble Aβ. The remaining pellet was re-homogenized with TBS containing 1% Triton-X (TBS-T) and centrifuged for 1 h at 16,000 × g at 4 °C to generate a soluble membrane-bound brain Aβ extract. Finally, the remaining pellet was homogenized with 70% formic acid (FA) and centrifuged at for 1 h at 16,000 × g at 4 °C to generate a brain extract of insoluble Aβ. The generated brain extracts were stored at − 80 °C until further application.

### Immunohistochemistry

Sagittal brain cryo-sections, 20 µm thick (Cryostar NX70 cryostat, Thermo Fisher Scientific), of the right hemisphere were fixated for 30 min at RT with cold acetone and treated with citrate buffer (pH 6) for antigen retrieval. The sections were then treated with 0.3% H_2_O_2_ (Sigma Aldrich, Stockholm, Sweden) to block endogenous peroxidase activity. This was followed by washing the sections for 5 min at RT with 0.4% Tween-20 in PBS to enhance tissue permeabilization. The sections were then blocked with 2.5% goat serum (Victorlabs, CA, USA) for 1 h at RT. The N-terminal specific anti-Aβ antibody 82E1, final concentration 1 µg/mL (IBL, Hamburg, Germany), was used as the primary antibody and the sections were incubated overnight at 4 °C. The following day, slides were washed with PBS, incubated with goat anti-mouse conjugated with Alexa-647 fluorophore for 1 h (Thermo Scientific, Waltham, MA), and washed with PBS. Following one wash with dH_2_O, the slides were mounted using a Vectashiled mounting medium (Victorlabs, CA) containing DAPI for nuclear staining.

### Aβ quantification ELISA

Ninety-six-well half-area plates (Corning Inc., Corning, NY) were coated overnight at 4 °C with 1 μg/mL of one of the following antibodies: 3D6 antibody binding to the N-terminal end of Aβ (recombinantly produced in the lab), m266 antibody binding to the mid-region of Aβ (recombinantly produced in the lab), A11 antibody binding to oligomers with beta hairpin formation (not exclusive to Aβ) (AHB0052, Thermo Scientific, Waltham, MA), mAb158 (recombinantly produced in the lab), mouse Aβ-specific antibody (Clone M3:2, 805,701, Nordic Biosite) or rabbit polyclonal antibodies detecting the C-terminal of Aβ (44–136, Thermo Scientific, Waltham, MA). If one wants to detect only aggregates in an ELISA, monoclonal antibodies that bind to the same place should be used as both coat and capture since in monomers this epitope will be blocked. Following 3-h incubation with ELISA blocking buffer (1% BSA in PBS), brain extracts and plasma samples were diluted in ELISA incubation buffer (0.1% BSA, 0.05% Tween-20 in PBS) and added to the plates. The plates were incubated overnight at 4 °C. This was followed by the addition of either rabbit polyclonal antibody detecting Aβ42 (700,254, Thermo Scientific, Waltham, MA) or biotinylated 3D6 (recombinantly produced in the lab) or biotinylated mAb27 (an arctic-Aβ selective antibody) [54] as detection antibodies. The plates were then incubated under agitation for 3 h at RT, followed by incubation with either Streptavidin-HRP (Mabtech, Stockholm, Sweden) or with a goat anti-rabbit HRP-conjugated antibody (AP307P, emdmillipore, Darmstadt, Germany) for additional 2 h at RT. Signals were developed as described above.

### Statistical analysis

Results are presented as mean ± SD (except AUC results that are presented as mean ± SEM). For detection of statistically significant differences in the results, data were analysed with either an unpaired t-test or one-way ANOVA followed by Bonferroni’s post hoc analysis. Significant *p*-values are defined as *p < 0.05, **p < 0.01 and ***p < 0.001.

## Results

### Protein design

**Fig. 1:**
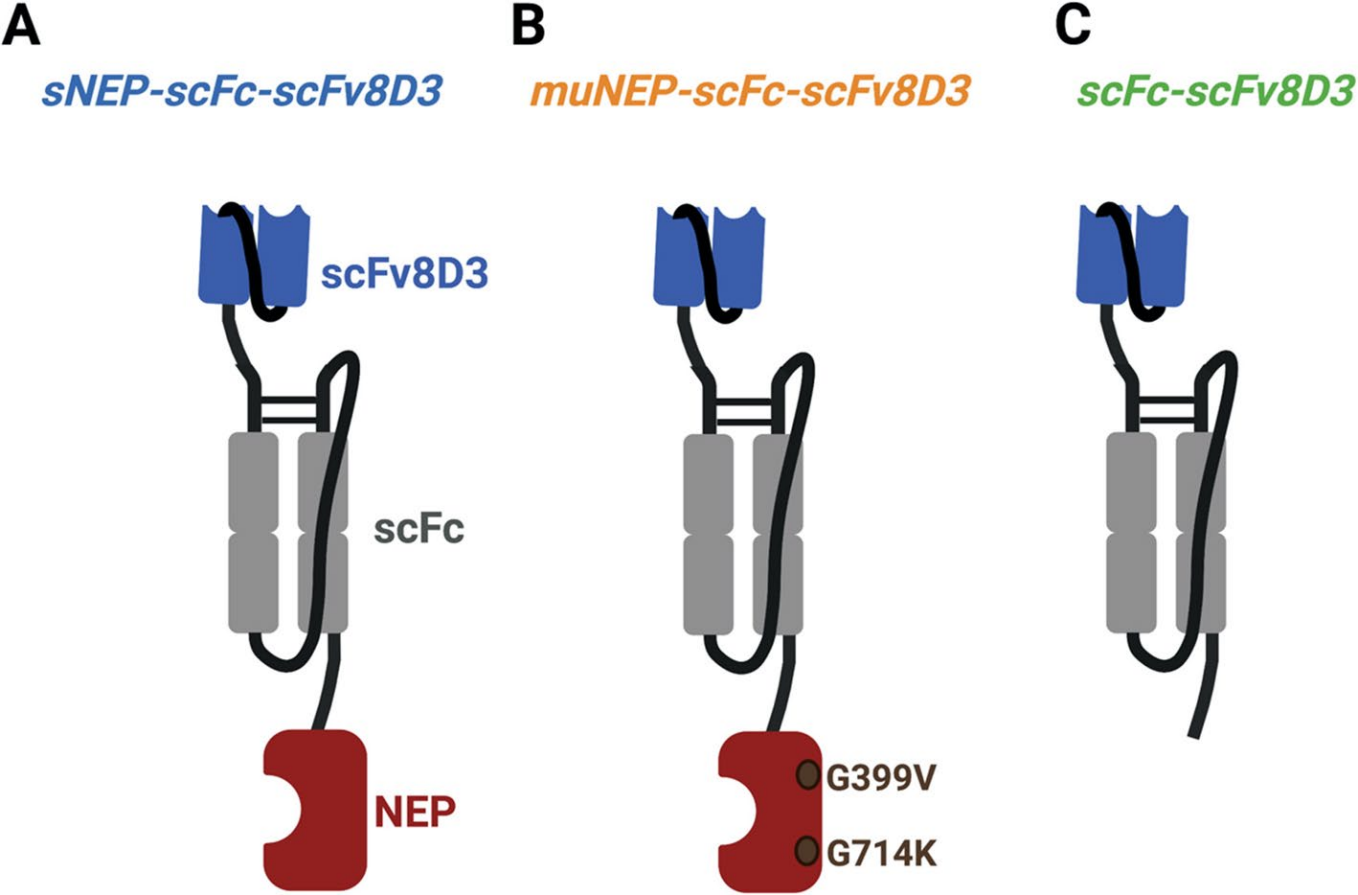
Design of the three recombinant proteins. **A** sNEP-scFc-scFv8D3, where the soluble (extracellular domain) of NEP is recombinantly linked to a scFc of mouse IgG2c antibody. The scFc is then linked to scFv8D3 (BBB transporter). **B** muNEP-scFc-scFv8D3, where a mutated variant of NEP (having higher degradation capacity on Aβ over other NEP substrates) is recombinantly linked to the scFc and scFv8D3 moieties as described above. **C** scFc-scFv8D3, where scFc is recombinantly linked to scFv8D3. This protein lacks NEP and is used as a control throughout the study. The three protein domains (NEP, scFc and scFv8D3) were recombinantly linked using APGSGGGSGPA linkers. The two parts of scFc were linked using the P(G_4_S)_3_ linker. The two parts of scFv8D3 were linked using the (G_4_S)_3_ linker

sNEP-scFc-scFv8D3 was generated by recombinantly linking sNEP (amino acid 52–749 of NEP) to a single-chain fragment constant (scFc) of mouse IgG2c antibody, which was attached to the BBB transporter (scFv8D3) (Fig. 1A) [17, 37, 38, 55]. The three proteins (NEP, scFc and scFv8D3) were linked together using an in-house designed linker (APGSGGGSGPA). The scFc was added to provide a long half-life and consisted of the CH2 and CH3 parts of mouse IgG2c antibody linked to another CH2-CH3 via P(G_4_S)_3_ linker (Morrison et al., 2022, submitted [56]). The second protein (muNEP-scFc-scFv8D3) was designed similar to the above-described protein, with the exception that soluble NEP was replaced with a mutated NEP variant including two-point mutations (G399V/G714K) (Fig. 1B). The third protein (scFc-scFv8D3) lacked NEP and consisted of scFc recombinantly linked to scFv8D3 as described above and was used as a control throughout the study (Fig. 1C).

### In vitro characterization of the recombinant proteins

**Fig. 2:**
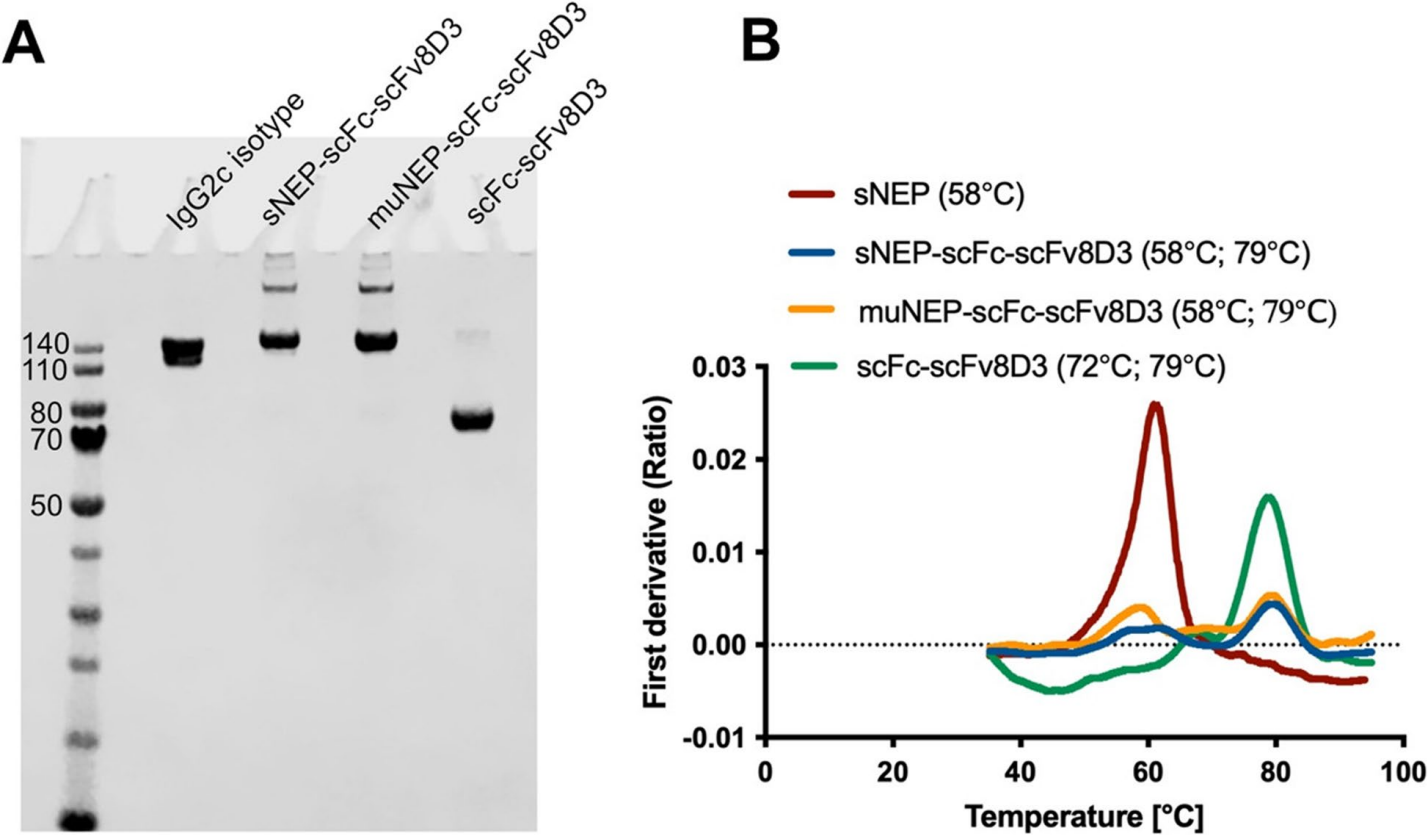
Purity analysis and structural stability of the recombinant proteins. **A** SDS-PAGE showing a strong band at around 160 kDa for sNEP-scFc-scFv8D3 and muNEP-scFc-scFv8D3 and a single band at around 80 kDa for scFc-scFv8D3. IgG2c isotype antibody of 150 kDa was loaded as a control. The complete SDS-PAGE gel can be seen in Supplementary Fig. 6. **B** Structural stability of the recombinant proteins under thermal stress measured with Tycho nt.6 system. The following inflection temperatures were detected for the proteins (58 °C for sNEP; 58 and 79 °C for sNEP-scFc-scFv8D3; 58 and 79 °C for muNEP-scFc-scFv8D3; 72 and 79 °C for scFc-scFv8D3)

The three proteins were recombinantly expressed in mammalian cell cultures [48], giving yields of 2.5–3.5 mg/L of transfection culture. SDS-PAGE analysis displayed a single band at approximately 80 kDa for scFc-scFv8D3 (Fig. 2A). A strong band at approximately 160 kDa (slightly bigger than that of mouse IgG2c antibody of 150 kDa) was detected for both sNEP-scFc-scFv8D3 and muNEP-scFc-scFv8D3. However, bigger bands were also seen for these two proteins, indicating the possible formation of protein multimers (Fig. 2A). Structural stability of the proteins was evaluated under thermal stress as described previously [49]. sNEP yielded an inflection temperature of 58 °C (Fig. 2B). sNEP-scFc-scFv8D3 yielded two inflection temperatures (58 and 79 °C) (Fig. 2B). The inflection temperature at 58 °C is attributed to the unfolding of NEP, while the one at 79 °C is likely related to the unfolding scFc of the IgG2c antibody [49]. muNEP-scFc-scFv8D3 yielded similar inflection temperatures as sNEP-scFc-scFv8D3 (Fig. 2B). The inflection temperatures for scFc-scFv8D3 were 72 °C and 79 °C (Fig. 2B).

### TfR and Aβ binding of the NEP-based recombinant proteins

**Fig. 3:**
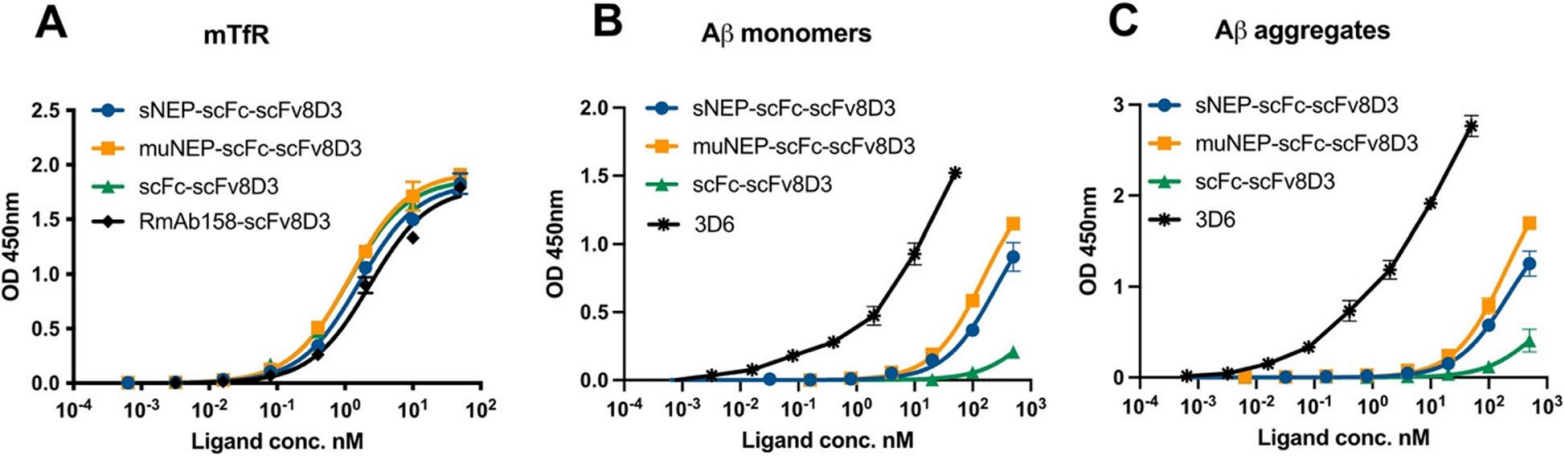
ELISA experiments displaying binding of the recombinant proteins to mouse TfR and Aβ. **A** Indirect ELISA demonstrating similar binding to mouse TfR among the recombinant proteins using RmAb158-scFv8D3 as the positive control. **B**, **C** Sandwich ELISA displaying similar binding of sNEP-scFc-scFv8D3 and muNEP-scFc-scFv8D3 scFv8D3 to Aβ1-40 monomers (**B**) and Aβ1-42 soluble aggregates (**C**). scFc-scFv8D3 that lacks Aβ biding domain displayed no binding to Aβ. Results presented as mean ± SD (*n* = 2). Data generated from two repetitive experiments

Binding of the recombinant proteins to mouse TfR was assessed with ELISA, using the previously described RmAb158-scFv8D3 antibody as a positive control [38]. Similar TfR binding was detected among the proteins (Fig. 3A). Furthermore, binding of the NEP-based proteins to Aβ1-40 monomers and Aβ1-42 aggregates was studied using scFc-scFv8D3 as the negative control and 3D6 antibody as the positive control. 3D6 has a comparable binding strength to both monomers and soluble aggregates of Aβ [57]. No significant differences were detected between sNEP-scFc-scFv8D3 and muNEP-scFc-scFv8D3 in binding to Aβ monomers and aggregates (Fig. 3B, C). Both NEP proteins displayed 50 times weaker binding to Aβ monomers and 100 times weaker binding to the aggregates compared to the 3D6 antibody control (Fig. 3B, C).

### Aβ degradation capacity of the recombinant proteins in vitro

**Fig. 4:**
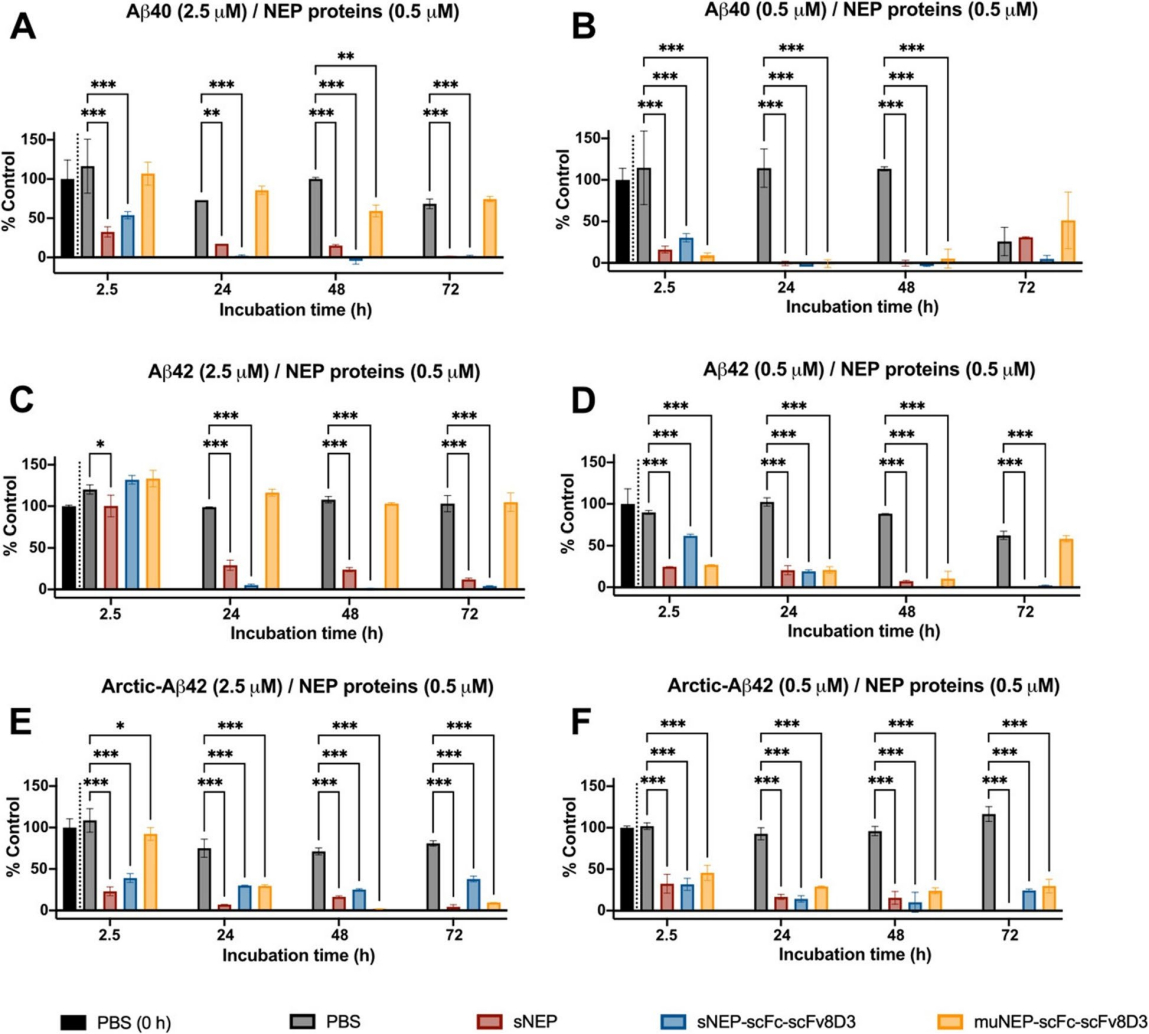
Degradation of Aβ in vitro by recombinant NEP proteins. Wt-Aβ1-40, wt-Aβ1-42 or arctic-Aβ1-42 peptides incubated with PBS, sNEP, sNEP-scFc-scFv8D3 or muNEP-scFc-scFv8D3 for 2.5, 24, 48 or 72 h at 37 °C. Degradation of Aβ measured in the different mixtures using sandwich ELISA that detects both monomers and aggregates. Two concentration ratios of Aβ/NEP proteins were used. **A** Aβ1-40 2.5 μM/NEP proteins 0.5 μM. **B** Aβ1-40 0.5 μM/NEP proteins 0.5 μM. **C** Aβ1-42 2.5 μM/NEP proteins 0.5 μM. **D** Aβ1-42 0.5 μM/NEP proteins 0.5 μM. **E** Arctic Aβ1-42 2.5 μM/NEP proteins 0.5 μM. **F** Arctic Aβ1-42 0.5 μM/NEP proteins 0.5 μM. Data are normalized to the Aβ/PBS treatment at 0 h. Results presented as mean ± SD. One-way ANOVA with Bonferroni’s multiple comparison test was applied (*n* = 2) (*p* > 0.05 = ns; *p* ≤ 0.05 = *; *p* ≤ 0.01 = **; *p* ≤ 0.001 = ***)

The ability of the recombinant proteins containing NEP to degrade Aβ was assessed in vitro using sNEP (NovusBio, Centennial, CO) as a positive control and PBS as a negative control. Recombinant NEP proteins were co-incubated at 37 °C with either Aβ1-40 or Aβ1-42 monomers. Degradation of Aβ was then assessed using a sandwich ELISA setup that only detects full-length Aβ (but both monomers and aggregates) and excludes the detection of fragmented Aβ. Two ratios of Aβ/NEP proteins were used: either a 5:1 ratio (2.5 μM of Aβ and 0.5 μM of the recombinant NEP proteins) or a 1:1 ratio (0.5 μM of Aβ and 0.5 μM of the recombinant NEP proteins). Both concentrations and ratios were different from what was used in the original muNEP paper [44]. Both sNEP (positive control) and sNEP-scFc-scFv8D3 were capable of degrading Aβ1-40 under both conditions (Fig. 4A, B). However, muNEP-scFc-scFv8D3 displayed Aβ1-40 degradation capacity only at 1:1 ratio (Fig. 4A, B). The same Aβ degradation patterns were detected for the Aβ1-42 isoform (Fig. 4C, D); however, the complete degradation of this isoform was slower compared to Aβ1-40 degradation for all NEP proteins. The assay was also performed using arctic-Aβ1-42 peptide. This Aβ peptide has a point mutation in the mid-region (E22G) resulting in facilitated formation of oligomers and protofibrils [46]. All NEP-based proteins were capable of degrading arctic-Aβ1-42 (Fig. 4E, F).

### Brain delivery and biodistribution of sNEP-scFc-scFv8D3 given at tracer doses

**Fig. 5:**
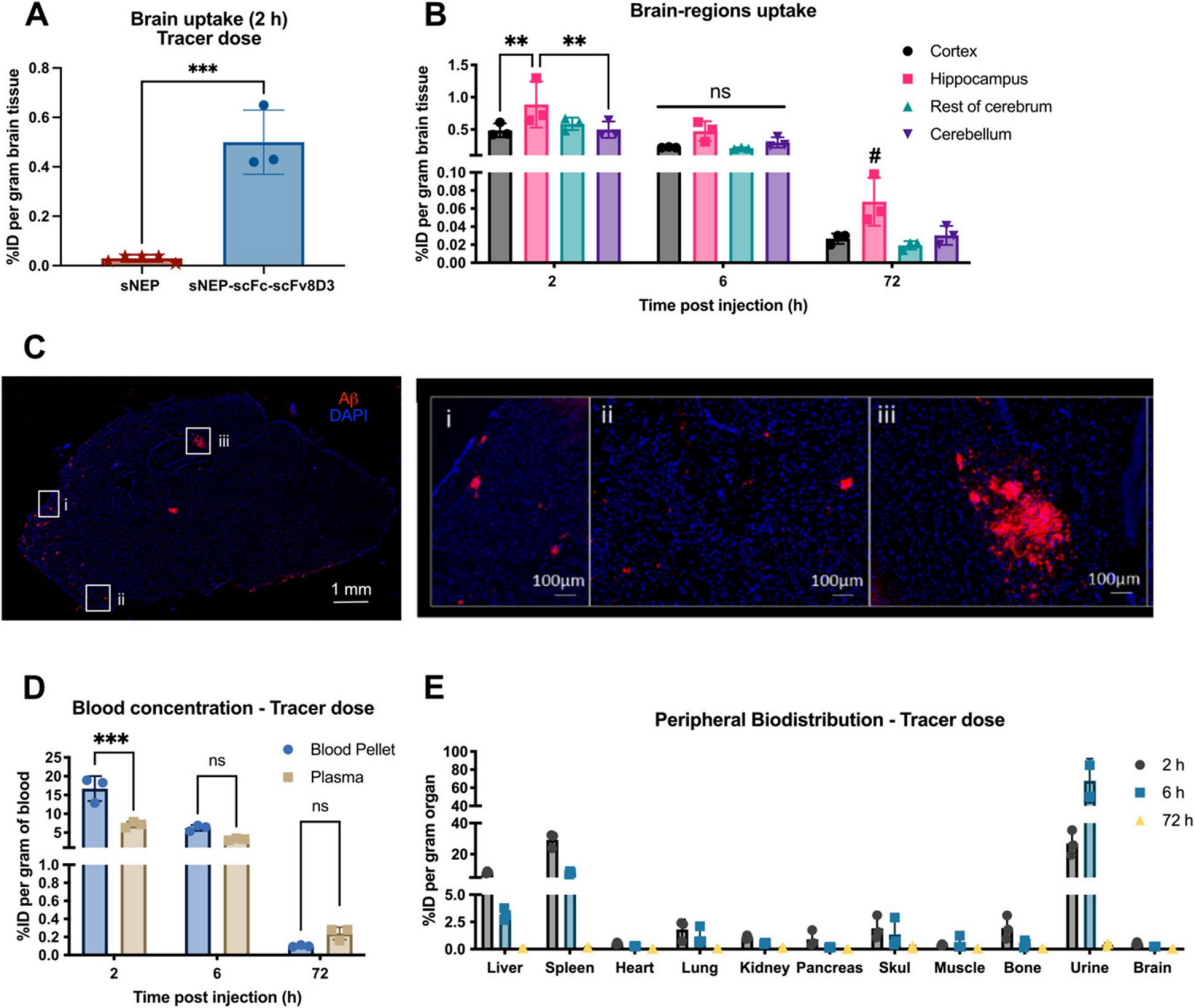
[^125^I]sNEP-scFc-scFv8D3 in the brain, blood and peripheral organs post tracer dose 0.05 mg/kg body weight/0.3 nmol/kg body weight administrated intravenously in the tail vein in 7–8-month-old tg-ArcSwe mice. **A** Significantly higher brain uptake of [^125^I]sNEP-scFc-scFv8D3 compared to [^125^I]sNEP at 2 h post-injection. The concentration of the radioactivity detected in the tissue is presented as percent of injected dose per gramme tissue (%ID/g). **B** Significantly higher uptake of [^125^I]sNEP-scFc-scFv8D3 in the hippocampus at 2 h post-injection compared to other brain regions. A trend towards a higher retention (#) in the hippocampus detected 72 h post-injection. **C** Aβ immunostaining of sagittal brain sections prepared from brains isolated at 72 h post-[^125^I]-sNEP-scFc-scFv8D3 administration to tg-ArcSwe mice. Boxed areas show Aβ immunostaining (red) and DAPI (blue) in the (i) cortex, (ii) ventral striatum and (iii) hippocampus. **D** Significantly higher amounts of [^125^I]sNEP-scFc-scFv8D3 in blood pellet compared to plasma 2 h post-injection. **E** Biodistribution of [.^125^I]sNEP-scFc-scFv8D3 in peripheral organs of tg-ArcSwe mice, at 2, 6 and 72 h post-injection. Results presented as mean ± SD. Unpaired *t-*test or one-way ANOVA with Bonferroni’s multiple comparison test were applied (*n* = 3/sNEP-scFc-scFv8D3; *n* = 5/sNEP) (*p* > 0.05 = ns; *p* ≤ 0.05 = *; *p* ≤ 0.01 = **; *p* ≤ 0.001 = ***)

Since NEP is likely to degrade Aβ monomers and small aggregates more efficiently [58], transgenic AD mice (tg-ArcSwe) of 7–8 months with emerging brain Aβ accumulation were used. Tg-ArcSwe mice at this age fairly young age have a higher proportion of soluble Aβ aggregates in comparison to insoluble plaques than older mice [54]. Mice were intravenously injected with tracer doses of 0.05 mg/kg body weight of [^125^I]sNEP or [^125^I]sNEP-scFc-scFv8D3. Brain uptake of sNEP-scFc-scFv8D3, expressed as %ID per gram brain tissue, was around 0.5% (Fig. 5A), which was 20-fold higher than the brain uptake of sNEP (Fig. 5A). Quantification of sNEP-scFc-scFv8D3 in different brain areas showed higher hippocampal concentrations at 2 h post-injection compared to the cortex and cerebellum (Fig. 5B). Concentration of sNEP-scFc-scFv8D3 drastically decreased in all brain regions 72 h post-injection (Fig. 5B). No significant differences were detected among the brain regions in the later time points, although hippocampal concentrations tended to be somewhat increased compared to what was observed in the other regions (Fig. 5B). The higher signals detected in the hippocampus may be attributed to the relatively high Aβ immunostaining detected in the hippocampus of 7–8 month-old tg-ArcSwe mice (Fig. 5C). The concentration of sNEP-scFc-scFv8D3 was also determined in the blood of the treated mice (Fig. 5D). Blood was separated into blood pellet (containing the blood cells) and plasma by centrifugation. Higher concentrations of the injected protein were detected in the blood pellet 2 h post-injection (Fig. 5D), similar to other scFv8D3-fused proteins [55]. Finally, the biodistribution of intravenously injected sNEP-scFc-scFv8D3 in different organs was studied. The spleen and liver represented organs with high concentrations of sNEP-scFc-scFv8D3 (Fig. 5E). However, the concentration of sNEP-scFc-scFv8D3 decreased substantially in almost all organs 72 h post-injection compared to the earlier time points (Fig. 5E).

Brain retention and blood pharmacokinetics of the recombinant proteins given at therapeutic doses.

**Fig. 6:**
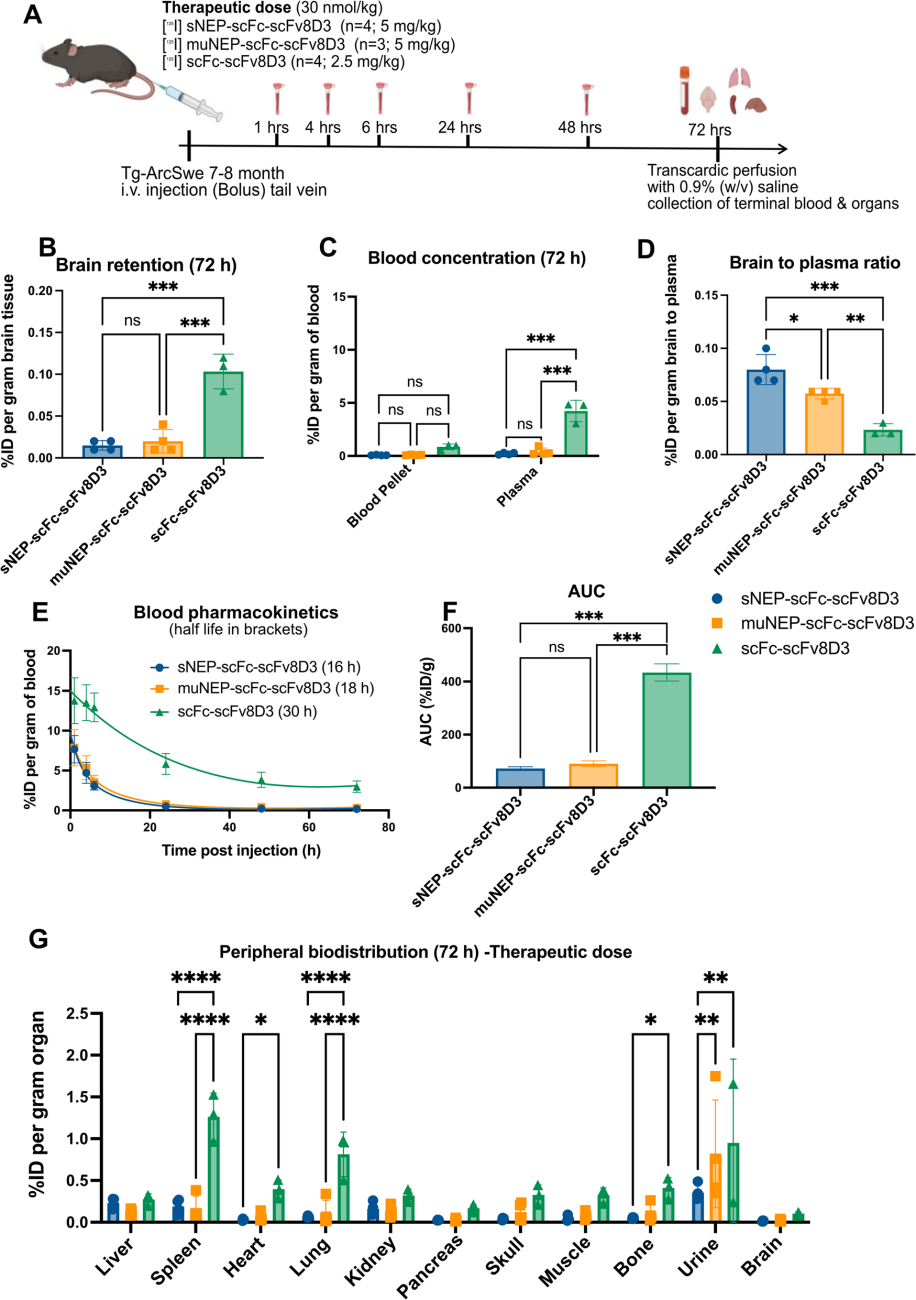
Concentrations in the brain, blood and peripheral organs after administration of [^125^I]sNEP-scFc-scFv8D3, [^125^I]muNEP-scFc-scFv8D3 and [^125^I]scFc-scFv8D3 at therapeutic doses of 30 nmol/kg body weight to 7–8 month-old tg-ArcSwe mice. **A**. Graphical figure of the experimental setup. **B** Significantly lower brain retention of [^125^I]sNEP-scFc-scFv8D3 and [^125^I]muNEP-scFc-scFv8D3 compared to [^125^I]scFc-scFv8D3 at 72 h post-injection. **C** Significantly lower plasma concentrations of [^125^I]sNEP-scFc-scFv8D3 and [^125^I]muNEP-scFc-scFv8D3 compared to [^125^I]scFc-scFv8D3, 72 h post-injection. **D** Brain-to-plasma concentration ratio of the three recombinant proteins at 72 h post-injection which illustrates transport to and from the brain. **E** Blood pharmacokinetics of the three injected proteins. Shorter half-lives were detected for [^125^I]sNEP-scFc-scFv8D3 and [^125^I]muNEP-scFc-scFv8D3 compared to [^125^I]scFc-scFv8D3. Values given in brackets. **F** Total drug exposure in blood, expressed as the area under the curve (AUC) of the three injected proteins. Fivefold lower blood exposure for [^125^I]sNEP-scFc-scFv8D3 and [^125^I]muNEP-scFc-scFv8D3 compared to [.^125^I]scFc-scFv8D3. **G** Biodistribution of the three injected proteins in peripheral organs at 72 h post-injection. Results are presented as mean ± SD (mean ± SEM for AUC results in **E**). One-way ANOVA with Bonferroni’s multiple comparison test was applied (*n* = 4/sNEP-scFc-scFv8D3 and muNEP-scFc-scFv8D3; *n* = 3/scFc-scFv8D3) (*p* > 0.05 = ns; *p* ≤ 0.05 = *; *p* ≤ 0.01 = **; *p* ≤ 0.001 = ***)

A therapeutic dose of 5 mg/kg body weight of [^125^I]sNEP-scFv8D3-scFc or [^125^I]muNEP-scFv8D3-scFc (corresponding to around 30 nmol/kg body weight; Mw 162 kDa) or 2.5 mg/kg body weight of [^125^I]scFv8D3-scFc (corresponding to around 30 nmol/kg body weight; Mw 81 kDa) was administered to 7.5–8 month-old tg-ArcSwe mice. Only 10% of the injected doses were labelled with [^125^I]; the rest was unlabelled. Significantly lower brain retention was observed for sNEP-scFc-scFv8D3 and muNEP-scFc-scFv8D3 compared to scFc-scFv8D3, 72 h post-injection (Fig. 6B). Interestingly, the differences were even larger in blood, as 40-fold lower plasma concentrations were detected for sNEP-scFc-scFv8D3 and muNEP-scFc-scFv8D3 compared to scFc-scFv8D3 (Fig. 6C). The brain-to-plasma ratio determines the concentration of the injected proteins in the brain compared to their concentration in plasma and is a measure of transport across the BBB. A higher brain-to-plasma ratio was detected for NEP-containing proteins compared to scFc-scFv8D3 (Fig. 6D), but since there is much more scFc-scFv8D3 in the plasma this will not inform about the total concentration in the brain. Blood half-life of the three recombinant proteins was calculated by measurement of radioactivity in blood samples collected from the tail vein at 1, 4, 6, 24, 48 and 72 h post-administration. Elimination half-life was estimated to 16 h and 18 h for sNEP-scFc-scFv8D3 and muNEP-scFc-scFv8D3, respectively (Fig. 6E). The blood concentrations of the two scFv8D3 fused NEP proteins were not different at any of the time points (Fig. 6E). A twofold longer blood half-life was calculated for scFc-scFv8D3 (Fig. 6E). Furthermore, the total drug exposure in blood, quantified by the area under the curve (AUC), of the scFv8D3-fused NEP proteins was 5 times lower than that of scFc-scFv8D3 (Fig. 6F). Finally, a significantly lower retention was observed for sNEP-scFc-scFv8D3 and muNEP-scFc-scFv8D3 compared to scFc-scFv8D3 in the spleen, lung and liver at 72 h post-injection (Fig. 6G).

It is possible that the radioactive signal that is measured does not come from the constructs but from free iodine that has been released if the proteins might be degraded. In order to ensure that this is not the case, an instant thin layer chromatography medium (iTLC) can be run. We did this before injection of the proteins as well as with urine and plasma after the in vivo experiments were finalized. The detected signal for the labelled proteins before injection and in plasma (Supplementary Fig. 2A & B) corresponds to the intact proteins whereas the detected signal in urine corresponds to free iodine (Supplementary Fig. 2 C).

### Aβ degradation capacity of the recombinant proteins in vivo.

**Fig. 7:**
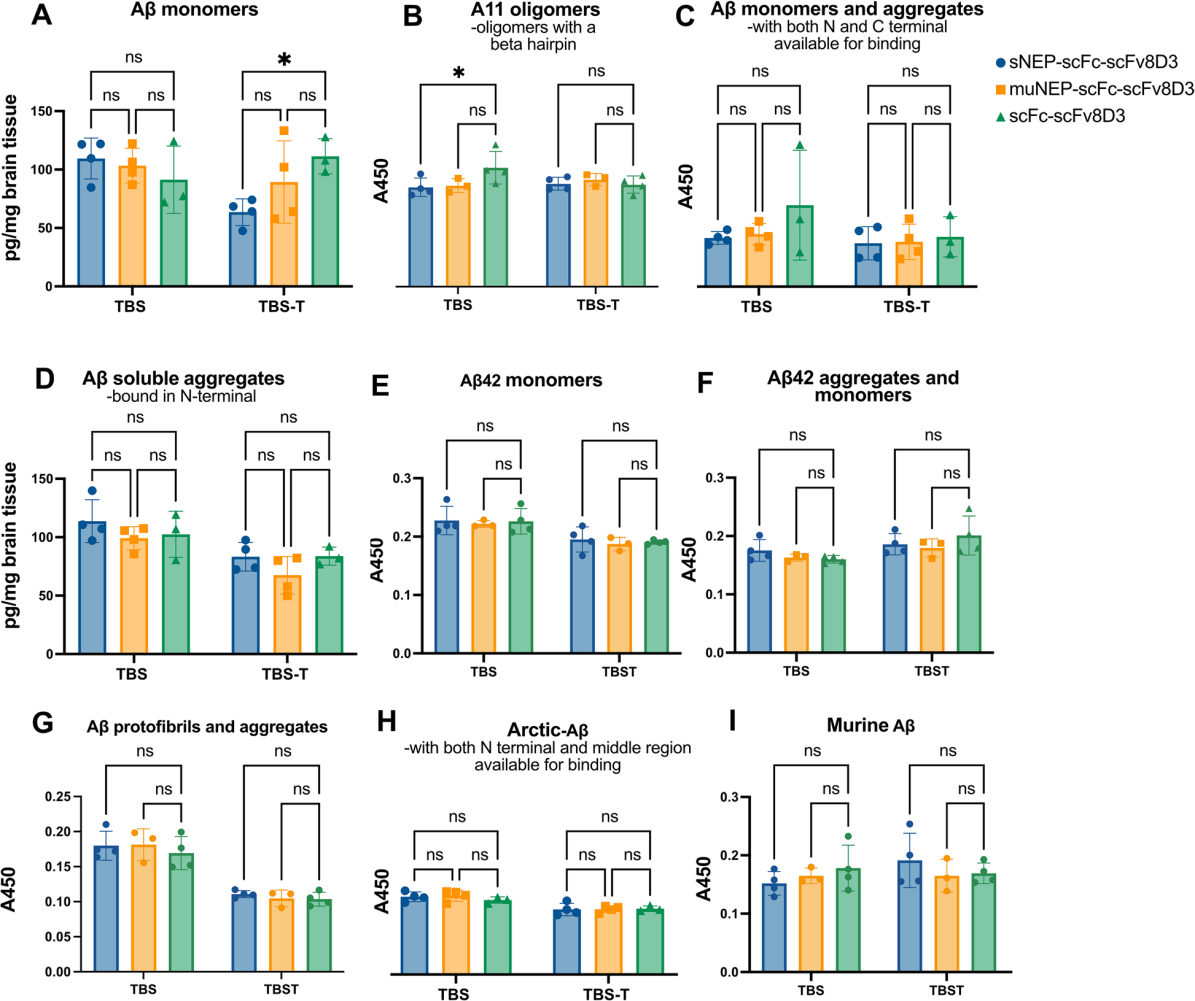
Concentration of Aβ in spun TBS and TBS-T extracts from the brain of tg-ArcSwe mice following treatment with therapeutic doses of 30 nmol/kg body weight of sNEP-scFc-scFv8D3 or muNEP-scFc-scFv8D3, using scFc-scFv8D3 as the negative control. **A** Significantly lower concentration of Aβ monomers (*p* value 0.03) was detected in the TBS-T brain extracts of the sNEP-scFc-scFv8D3-treated group compared to the scFc-scFv8D3 control group using a m266-3D6 ELISA. **B** Also, the levels of oligomers with a hairpin were significantly lower after treatment detected with a A11-3D6 ELISA. **C** Using an ELISA with one antibody detecting the N-terminal (3D6) and one detecting the C-terminal of Aβ, i.e. an ELISA that will detect aggregates with no hidden terminal and monomers, no significant differences were detected. **D** ELISA using the monoclonal antibody 3D6 as both capture and detection which will exclude the detection of monomers and will also allow the detection of aggregates that have the C-terminal hidden. **E** m266-Aβ42 ELISA. **F** 3D6-Aβ42 ELISA. **G** mAb158-3D6 ELISA. **H** ELISA with the 3D6 antibody as capture and the arctic-specific mAb27 antibody as detection. **I** Murine Aβ-3D6 ELISA. Results are presented as mean ± SD. One-way ANOVA with Bonferroni’s multiple comparison test was applied (*n* = 4/sNEP-scFc-scFv8D3 and muNEP-scFc-scFv8D3; *n* = 3/scFc-scFv8D3) (*p* > 0.05 = ns; *p* ≤ 0.05 = *; *p* ≤ 0.01 = **; *p* ≤ 0.001 = ***). Standard curves were used when possible. In cases of detection of a mixture of aggregates and monomers, no standard curve would be able to correctly correspond to the levels measured in homogenates and therefore the A450 measured is given, which cannot be compared between different ELISAs

Post-mortem brain tissues from tg-ArcSwe mice administered with therapeutic doses of sNEP-scFc-scFv8D3, muNEP-scFc-scFv8D3 or scFc-scFv8D3 were homogenized to generate brain extracts of soluble (TBS), membrane-bound (TBS-T) and insoluble (FA) Aβ. The concentration of Aβ in the three extracts was quantified using different ELISA setups. As mentioned previously, tg-ArcSwe mice have both arctic-Aβ (from the transgene) in the brain and wt-Aβ (mouse Aβ) both in the brain and blood. For the quantification of Aβ monomers, the m266 antibody (murine version of Solanezumab) was used as the capture antibody. m266 binds to an epitope in the mid-region of Aβ (aa 16–26) [59] and binds equally well to wt-Aβ and arctic-Aβ (Supplementary Fig. 3). Using an inhibition ELISA, we demonstrated a strong binding of m266 to Aβ monomers compared to aggregates (Supplementary Fig. 4). The binding strength of m266 decreased drastically with the increase in the size of Aβ aggregates (Supplementary Fig. 4). When using m266-based ELISA, a significantly lower concentration (*p* value 0.03) of membrane-bound (TBS-T) monomeric Aβ was detected in mice treated with sNEP-scFc-scFv8D3 compared to the control group treated with scFc-scFv8D3 (Fig. 7A). The most likely most toxic Aβ species are the small oligomers that contain a beta hairpin (that is converted to beta sheets in larger aggregates). This beta hairpin can be detected by the, by others well characterized, A11 antibody. The A11 antibody is not specific to Aβ beta hairpins but does also detect others like for instance alpha-synuclein. In the A11 ELISA, a significant reduction was detected in the TBS fraction after treatment with sNEP-scFc-scFv8D3 (Fig. 7B). Aggregates can fold in many different ways leaving different epitopes available for binding and detection. For the quantification of Aβ aggregates that have the N-terminal free, the 3D6 antibody was used as the capture antibody and it is in biotinylated form as the detection antibody. The use of the same antibody as the capture and detection antibody will exclude binding to monomers due to epitope self-blocking. Using such an ELISA setup, no differences were detected in the concentration of Aβ aggregates following the different treatments (Fig. 7D). Aggregates that have both the N- and C-terminal free can be detected with a sandwich ELISA using antibodies that bind to these sites. This ELISA will also detect monomers since there is no epitope self-blocking. No differences could be detected for these aggregates and monomers (Fig. 7C). To see if it was possible to detect which type of monomers that are reduced in the TBS-T, a monomeric Aβ42 ELISA was done, but no significant differences could be detected. Also, a protofibril ELISA was done using the murine version of Lecanemab. To selectively quantify arctic-Aβ, mAb27 was used as the detection antibody. This antibody is highly selective for arctic-Aβ (Supplementary Fig. 3). Such ELISA can detect both monomeric and aggregated arctic-Aβ. However, due to its high aggregation propensity, most of the arctic-Aβ is expected to be in the form of aggregates. No differences were detected in the concentration of arctic-Aβ following the different treatments (Fig. 7H). Finally, no differences were detected in the concentration of total mouse Aβ in TBS soluble and TBS-T soluble extracts among the three groups (Fig. 7I). FA-soluble brain extracts that contain insoluble Aβ were also analysed and, as expected, no significant differences were detected between the groups (Supplementary Fig. 5).

**Fig. 8:**
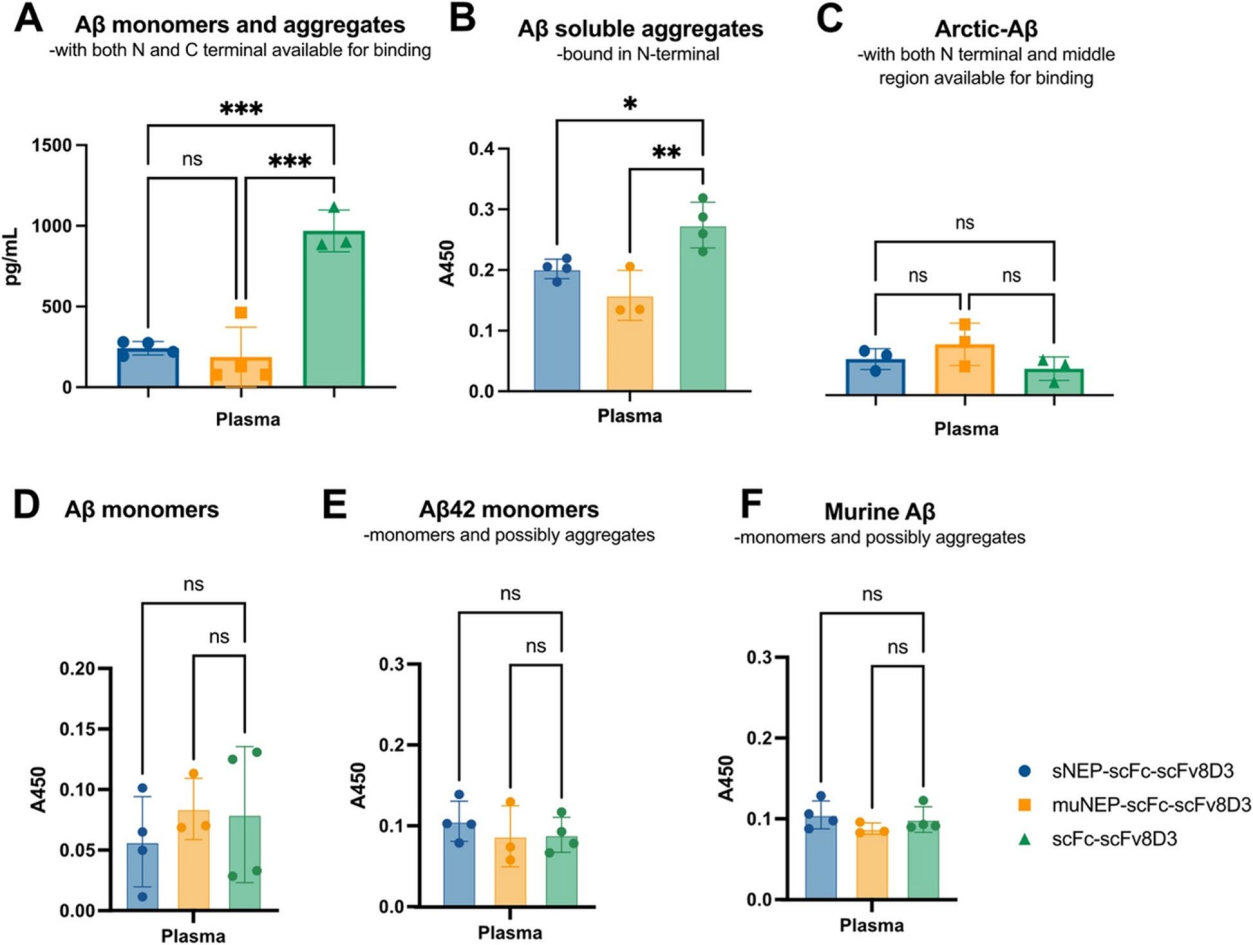
Concentration of Aβ in the plasma of heterozygote tg-ArcSwe mice following treatment with therapeutic doses of 30 nmol/kg body weight of sNEP-scFc-scFv8D3 or muNEP-scFc-scFv8D3, using scFc-scFv8D3 as the negative control. Significantly lower concentrations were detected of Aβ with free N and C-terminals (monomers and some of the aggregates) (**A**) and soluble aggregates with the N-terminal free (**B**) but not of Aβ detected with the arctic-specific antibody (middle region) and a N-terminal antibody (**C**). Since no difference was detected for monomers (**D**), or more specifically Aβ42 monomers, it seems like the difference is in the aggregates and possibly in the aggregates with the N- and C-terminal free. No difference was neither detected in murine Aβ. One-way ANOVA with Bonferroni’s multiple comparison test was applied (*n* = 3–4) (*p* > 0.05 = ns; *p* ≤ 0.05 = *; *p* ≤ 0.01 = **; *p* ≤ 0.001 = ***)

Since NEP likely is more effective in degrading monomers than large insoluble aggregates, and since monomers can be present at high concentrations in the periphery, we sought to quantify Aβ concentrations in the plasma of tg-ArcSwe mice treated with the recombinant proteins. To quantify total Aβ in plasma, a C-terminal anti-Aβ (that detects both Aβ40 and Aβ42) antibody was used as the capture antibody in an ELISA and 3D6 as the detection antibody. This ELISA will detect both monomers and aggregates of Aβ. Treatment of tg-ArcSwe mice with sNEP-scFc-scFv8D3 and muNEP-scFc-scFv8D3 gave rise to a significant reduction in the plasma concentration of monomers and aggregates Aβ of with the N–C-terminal free compared to the control group (Fig. 8A). These effects were evident also when quantifying the soluble aggregates with only the N-terminal free (Fig. 8B) but not for monomers or murine Aβ (that is likely not to aggregate) (Fig. 8D–F). The changes could not be seen using the arctic-specific ELISA that would detect aggregates and monomers that have the N-terminal and the middle region free.

## Discussion

NEP is a naturally occurring membrane-bound metallopeptidase that has gained a growing interest in the field of AD due to its ability to degrade Aβ. Several studies have suggested the use of recombinant NEP to generate therapeutic interventions in AD [27, 60]. However, similar to other large molecules, NEP is unable to reach intra-brain targets when peripherally administered due to the presence of the BBB. In the current study, we aimed at developing BBB-penetrant recombinant proteins based on NEP and testing their brain biodistribution and ability to degrade Aβ in a transgenic mouse model of AD.

For this purpose, the previously described and very efficient BBB transporter (scFv8D3) binding to mouse TfR [17, 38, 39, 61] was recombinantly linked to soluble NEP (sNEP) and an Fc-region fragment (scFc) of mouse IgG2c antibody, forming the final protein drug (sNEP-scFc-scFv8D3). In addition to the mentioned protein, we designed another recombinant protein (muNEP-scFc-scFv8D3) that was based on a mutated variant of NEP displaying lower efficiency in degrading other NEP substrates compared to Aβ [44].

Following purification, the two NEP proteins were present mainly in the monomeric form with a strong band in SDS-PAGE at around 160 kDa (Fig. 2A). However, SDS-PAGE analysis demonstrated some weaker bands that were bigger than 160 kDa, suggesting the formation of protein multimers. A previous study has demonstrated the dimerization of naturally occurring NEP [62], which could suggest that the formation of sNEP-scFc-scFv8D3 and muNEP-scFc-scFv8D3 multimers is related to the presence of NEP in these designs. Nevertheless, the possibility that the other domains such as scFc contributed to the formation of these multimers cannot be entirely excluded. A rather rapid degradation of the NEP constructs could be detected in vivo, a possible reason for this could be the propensity to form multimers which often leads to more rapid degradation.

The ability of sNEP-scFc-scFv8D3 to degrade Aβ was first assessed in vitro using sNEP as the positive control. In our designs, the N-terminal of sNEP is fused to other protein domains (scFc-scFv8D3). Therefore, we wanted to evaluate whether such protein engineering might interfere with the ability of NEP to degrade Aβ. sNEP-scFc-scFv8D3 preserved NEP properties in binding to and degrading Aβ monomers in vitro (Figs. 3 and 4). These results indicate that the recombinant addition of scFc and scFv8D3 on the N-terminal end of NEP is a suitable strategy to develop NEP-based protein drugs with long half-life and capability to cross BBB, respectively.

sNEP-scFc-scFv8D3 could, similar to sNEP, significantly degrade Aβ1-40 and Aβ1-42 monomers in vitro (Fig. 5) both at the 1:1 and 5:1 ratios. Surprisingly, the recombinant protein based on the mutated NEP protein (muNEP-scFc-scFv8D3) was not able to degrade wt-Aβ at a 5:1 ratio, where the concentration of Aβ was higher, 2.5 μM. Degradation was evident only when a 1:1 ratio of Aβ and NEP proteins was used and with a 0.5-μM concentration of Aβ (Fig. 4). The higher the concentration of Aβ, the faster the aggregation is a well-known fact. It is possible that the concentration of 2.5 μM was high enough to start aggregation already before the first time point analysed and that the muNEP is worse at degrading aggregates than sNEP. muNEP has been previously reported to exert a 20 times higher Aβ degradation capacity compared to wild-type NEP but this was at much higher concentrations of Aβ [44] and a much lower ratio than we had at other concentrations it seemed from the data that it was less effective. Wild-type NEP (sNEP in our study) has the capacity to degrade Aβ at several cleavage sites between amino acids 1 to 35. However, muNEP preferentially degrades Aβ at position 20–21 located at the mid-region of Aβ [44]. This region becomes gradually hidden with the formation of aggregates, further suggesting the inability of muNEP to degrade aggregated forms Aβ.

The in vitro Aβ degradation results (Fig. 4) demonstrated the ability of sNEP-scFc-scFv8D3 to efficiently degrade both Aβ1-40 and Aβ1-42 monomers. Interestingly, sNEP-scFc-scFv8D3 could degrade Aβ1-40 faster than Aβ1-42 at high Aβ concentrations (Fig. 4A–C). NEP cleavage sites are located within the first 35 amino acids of Aβ [63], meaning that the enzyme should have an equal degradation efficiency on both Aβ isoforms. NEP is more efficient in degrading monomers, and since Aβ1-42 is more prone to aggregate, it is more likely that this isoform started to aggregate during the experiment, hence slower degradation of this isoform compared to Aβ1-40. Our results are in agreement with [64], demonstrating NEP ability to degrade synthetic Aβ1-40 and Aβ1-42 peptides in vitro. Similar to our results, the mentioned study demonstrated NEP ability to degrade Aβ1-40 faster compared to Aβ1-42. On the contrary, we have recently demonstrated a selective degradation of Aβ42 (but not Aβ40) in the hippocampus of AD mice mediated by enhanced NEP expression following treatment with a brain-penetrating somatostatin peptide [17]. However, our previous study demonstrated the degradation efficiency of membrane-bound NEP, while the current study is based on the use of the soluble part of NEP (sNEP). These studies suggest NEP-mediated degradation of Aβ to be dependent on several factors such as the availability and concentration of Aβ, its aggregation state and the form of NEP (membrane-bound or soluble).

A bit to the contrary of the discussion above, both NEP constructs degraded the fast-aggregating arctic Aβ the best in the conditions used. A possible reason for this can be that arctic Aβ is known to form protofibrils but less of insoluble aggregates [46]. The structure of these protofibrils has not been determined, but they possibly retain the beta hairpin that is also present in oligomers and the first thing that forms when monomers start to aggregate. This hair pin converts to larger beta sheets in the larger aggregates. In the modelling of NEP binding site to Aβ, it seems like this hair pin still could be accessible while the beta sheets of the aggregates would not be. That is, it is possible that NEP can degrade the aggregates formed by arctic Aβ, but not other types of aggregates. That this effect is the strongest for muNEP could be due to that the altered binding of muNEP compared to NEP is exactly on the amino acid 22 which is the aa that is mutated in the arctic Aβ.

By recombinantly linking the TfR binding moiety (scFv8D3) to our constructs, we could detect high brain uptakes of the NEP protein drug in tg-ArcSwe mice. Brain uptake was 20 times higher compared to the sNEP protein lacking scFv8D3 (Fig. 5A), which is a similar increase as seen with other scFv8D3 fusions to large proteins [17, 39]. In addition, high concentrations were detected in the hippocampus (Fig. 5B), a brain area where Aβ pathology starts [45], and where strong Aβ immunostaining is detected in young tg-ArcSwe mice (Fig. 5C). We have previously also analysed how much of free iodine that enters the brain to see if degradation of constructs is likely to have an effect on the measured uptake but we see that very little free iodine can enter the brain and are therefore not likely to significantly contribute to the signal [65].

The addition of scFc domains prolonged the blood half-life of the NEP-based recombinant proteins compared to the short half-life of soluble NEP. Nevertheless, significantly lower plasma concentrations, twofold shorter blood half-life and fivefold lower drug exposure (AUC blood) of sNEP-scFc-scFv8D3 and muNEP-scFc-scFv8D3 were displayed compared to scFc-scFv8D3 (Fig. 6C–F). Binding of NEP present in these designs to Aβ, but also other NEP substrates, in the blood followed by subsequent degradation could be one of the possible reasons of the low concentrations of sNEP-scFc-scFv8D3 and muNEP-scFc-scFv8D3 detected in the blood. The high liver concentrations of sNEP-scFc-scFv8D3 at the early time points (Fig. 5E) further suggest protein degradation. In addition to the low concentrations in the blood, the brain concentrations of NEP proteins drastically decreased 72 h post-injection (Fig. 6B), opposite to what has been previously reported with other Aβ binders containing scFv8D3 [41], suggesting fast clearance of the NEP proteins from the brain. The same pattern of low NEP protein concentration was also evident in the lung and heart (Fig. 6G), two organs containing several NEP substrates [43, 66, 67].

The ability of the recombinant NEP proteins to degrade Aβ in vivo was investigated using tg-ArcSwe mice treated with a single therapeutic dose of 30 nmol/kg. sNEP-scFc-scFv8D3 and muNEP-scFc-scFv8D3 significantly decreased aggregated Aβ concentration in plasma but not of monomers (Fig. 8A, B). The significant reduction in aggregated plasma Aβ levels did not significantly affect the levels of Aβ aggregates, except oligomers in the brain (Fig. 7). In the brain, we saw a small but significant reduction of Aβ monomers in the TBS-T fraction and of Aβ oligomers as detected by the A11 antibody that detects oligomers with a beta hairpin in the TBS fraction. This beta hairpin is lost when the aggregates grow and form fibrils. The reduction we see in hairpin containing Aβ goes in line with the possibility that neprilysin is efficient in degrading hairpin containing oligomers as we discussed above. Since no significant reduction in the amount of Aβ42 monomers could be detected in the brain, we assume that it is Aβ40 that is reduced. It is also the main type of Aβ in the ArcSwe mice.

There is also a possibility that the effects seen in the brain are due to the reduction of Aβ in the blood, but since it is mainly a reduction in aggregates in the blood and not monomers, we think that is less likely.

Our findings are in accordance with previous studies using similar proteins consisting of recombinant NEP linked to either an albumin protein [68] or the Fc region of IgG antibodies [69]. NEP proteins used in these studies demonstrated no alteration in Aβ concentration in the brain despite the significant Aβ reduction detected in the periphery. However, none of these studies used a BBB transporter and was based on the sink hypothesis of Aβ equilibrium between the brain and periphery. In our study, we used scFv8D3 as the BBB transporter which facilitated the delivery of recombinant NEP proteins into the brain. In addition, treatment with sNEP-scFc-scFv8D3 was associated with a selective reduction (*p* value 0.03) in membrane-bound Aβ monomers, but not aggregates. These findings are similar to previous studies demonstrating more efficient degradation of Aβ monomers by NEP compared to oligomers [33].

The in vitro experiments demonstrated that NEP-based proteins were capable of degrading arctic-Aβ, opposite to what has been previously reported [70]. Based on the in vitro results, we chose the tg-ArcSwe AD mouse model for the in vivo experiments. Nevertheless, treatment with scFv8D3-fused NEP proteins failed to display any effects on the concentration of arctic-Aβ in tg-ArcSwe mice, but this can be attributed to the ELISA that is set up. With the ELISA used, one will only detect aggregates that have both the N-terminal and the middle available for binding, and likely mostly aggregates will be detected due to the coating with the 3D6 antibody that binds both aggregates and monomers, but that likely will favour aggregates due to the avidity effect. The short retention time of the injected NEP in the brain will likely reduce the possible effect the NEP have had in the brain.

### Limitations

One limitation can be the small sample size, especially in a treatment study where *n* = 3–4 per treatment group. However, since we see this study as a proof of concept demonstrating the treatment effects of BBB-penetrating formats of NEP, we followed the 3R principles and used the minimal number of animals needed to display a statistically significant effect.

## Conclusion

Overall, the present study shows the potential of using TfR-mediated transcytosis to successfully deliver NEP proteins into the brain. By adding a scFc and a scFv8D3, our new NEP-based protein designs displayed longer blood half-life and 20 times higher brain uptake compared to sNEP. The retained enzymatic activity in vitro and the significant reduction of Aβ monomers and Aβ oligomers in vivo following a single intravenous injection highlight the potential of recombinant NEP-based proteins in AD although a modified construct with better brain retention would be desired.

## Supplementary Information

**Additional file 1:** **Supplementary Figure 1.** The sequences of the genes that was inserted in to the pcDNA3.4 vectorused to produce the constructs. The signal peptide is cleaved while expressingthe protein so is not left in the final protein that have been used. A. sNEP-scFc-scFv8D3.B. muNEP-scFc-scFv8D3. C. scFc-scFv8D3. **Supplementary Figure 2.** Instant thin layer chromatography (iTLC) of proteinsbefore and after in vivo experiment. A. Proteins after labelling with ^125^I and before application in vivo study. B. Plasmasamples and C. Urine samples 72 hours post injection of 30 nmol/kg body weightof SNEP-scFcscFv8D3, muNEP-scFc-scFv8D3 and scFc-scFv8D, which was appliedintravenously in the tail vein. All samples were applied on a silica-coatedaluminium plate and separated with 70% (v/v) acetone. The radioactive signalwas developed with an X-ray film and red in a Cyclon Phosphoimager. **Supplementary Figure 3.**Binding selectivity of anti-Ab antibodies used in this study. ELISA plates coatedwith an anti-Ab42capture antibody. Serial dilution of either wild-type Ab1-42 (wt-Ab)or arctic-Ab1-42added to the plates. **A:** when using 3D6 as the detection antibody, bothwt-Aband arctic-Abcould be detected. **B:** when using m266 as the detection antibody, bothwt-Aband arctic-Abcould be detected. **C:** when using mAb27 as the detection antibody, onlyarctic-Abcould be detected. **Supplementary Figure 4.** Inhibition ELISA demonstrating the binding strengthof m266 antibody to different species of Ab.Five different Ab species were used: Ab1-40monomers, Ab1-40 dimers, Ab1-42oligomers, Ab1-42 protofibrils and Ab1-42fibrils, prepared as described previously [40]. The assay was performed as described previously [40]. Inhibitory concentration-50 (IC50) of m266 bindingto the different Ab species is present in the table. m266 bound strongerto Ab monomers compared to other Abspecies. Binding strength of m266 antibody decreased as the size of Abspecies increased. **Supplementary Figure 5. ** Total concentration of Ab40 and Ab42 in FA soluble brainextracts of tg-ArcSwe mice following treatment with therapeutic doses of 30nmol/kg body weight of sNEP-scFc-scFv8D3 or muNEP-scFc-scFv8D3, usingscFc-scFv8D3 as the negative control. No significant differences detected amongthe three groups. Results presented as mean ±SD. One-way ANOVA with Bonferroni’s multiple comparison test was applied(n=4/sNEP-scFc-scFv8D3 and muNEP-scFc-scFv8D3; n=3/scFc-scFv8D3).(*p*>0.05=ns; *p*≤0.05= *; *p*≤0.01= **; *p*≤0.001= ***). **Supplementary Figure 6.** Complete image of the SDS-PAGE gel presented in Fig.2A.

## Data Availability

The datasets used and/or analysed during the current study are available from the corresponding author on reasonable request.

## Abbreviations

Aβ: Amyloid-beta
AD: Alzheimer’s disease
APP: Amyloid precursor protein
Arc: Arctic APP mutation
BBB: Blood–brain barrier
muNEP: Mutated variant of neprilysin (G399V/G714K)
NEP: Neprilysin
scFc: Single-chain fragment constant
scFv: Single-chain fragment variable
sNEP: Soluble neprilysin
Swe: Swedish APP mutation
TfR: Transferrin receptor
WT: Wild-type
