# Supplementary material for: Blood–brain barrier penetrating neprilysin degrades monomeric amyloid-beta in a mouse model of Alzheimer’s disease
Source: Alzheimers Res Ther. 2022 Dec 5;14:180. doi: 10.1186/s13195-022-01132-2 (PMC9720954; doi:10.1186/s13195-022-01132-2)
Supplement: Supplementary file 1 — Additional file 1: Supplementary Figure 1. The sequences of the genes that was inserted in to the pcDNA3.4 vectorused to produce the constructs. The signal peptide is cleaved while expressingthe protein so is not left in the final protein that have been used. A. sNEP-scFc-scFv8D3.B. muNEP-scFc-scFv8D3. C. scFc-scFv8D3. Supplementary Figure 2. Instant thin layer chromatography (iTLC) of proteinsbefore and after in vivo experiment. A. Proteins after labelling with 125I and before application in vivo study. B. Plasmasamples and C. Urine samples 72 hours post injection of 30 nmol/kg body weightof SNEP-scFcscFv8D3, muNEP-scFc-scFv8D3 and scFc-scFv8D, which was appliedintravenously in the tail vein. All samples were applied on a silica-coatedaluminium plate and separated with 70% (v/v) acetone. The radioactive signalwas developed with an X-ray film and red in a Cyclon Phosphoimager. Supplementary Figure 3.Binding selectivity of anti-Ab antibodies used in this study. ELISA plates coatedwith an anti-Ab42capture antibody. Serial dilution of either wild-type Ab1-42 (wt-Ab)or arctic-Ab1-42added to the plates. A: when using 3D6 as the detection antibody, bothwt-Aband arctic-Abcould be detected. B: when using m266 as the detection antibody, bothwt-Aband arctic-Abcould be detected. C: when using mAb27 as the detection antibody, onlyarctic-Abcould be detected. Supplementary Figure 4. Inhibition ELISA demonstrating the binding strengthof m266 antibody to different species of Ab.Five different Ab species were used: Ab1-40monomers, Ab1-40 dimers, Ab1-42oligomers, Ab1-42 protofibrils and Ab1-42fibrils, prepared as described previously [40]. The assay was performed as described previously [40]. Inhibitory concentration-50 (IC50) of m266 bindingto the different Ab species is present in the table. m266 bound strongerto Ab monomers compared to other Abspecies. Binding strength of m266 antibody decreased as the size of Abspecies increased. Supplementary Figure 5. Total concentration of Ab4 [file 13195_2022_1132_MOESM1_ESM.docx]

**Supplementary information**

A


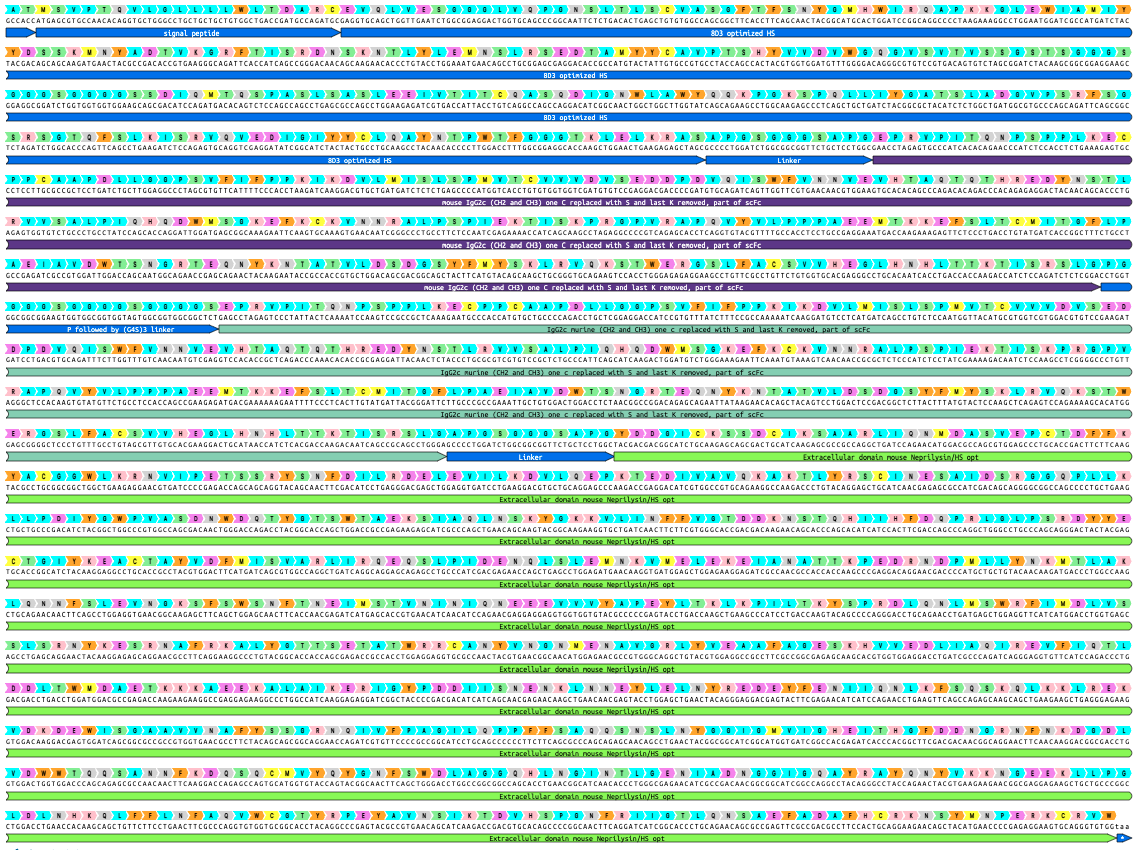


**
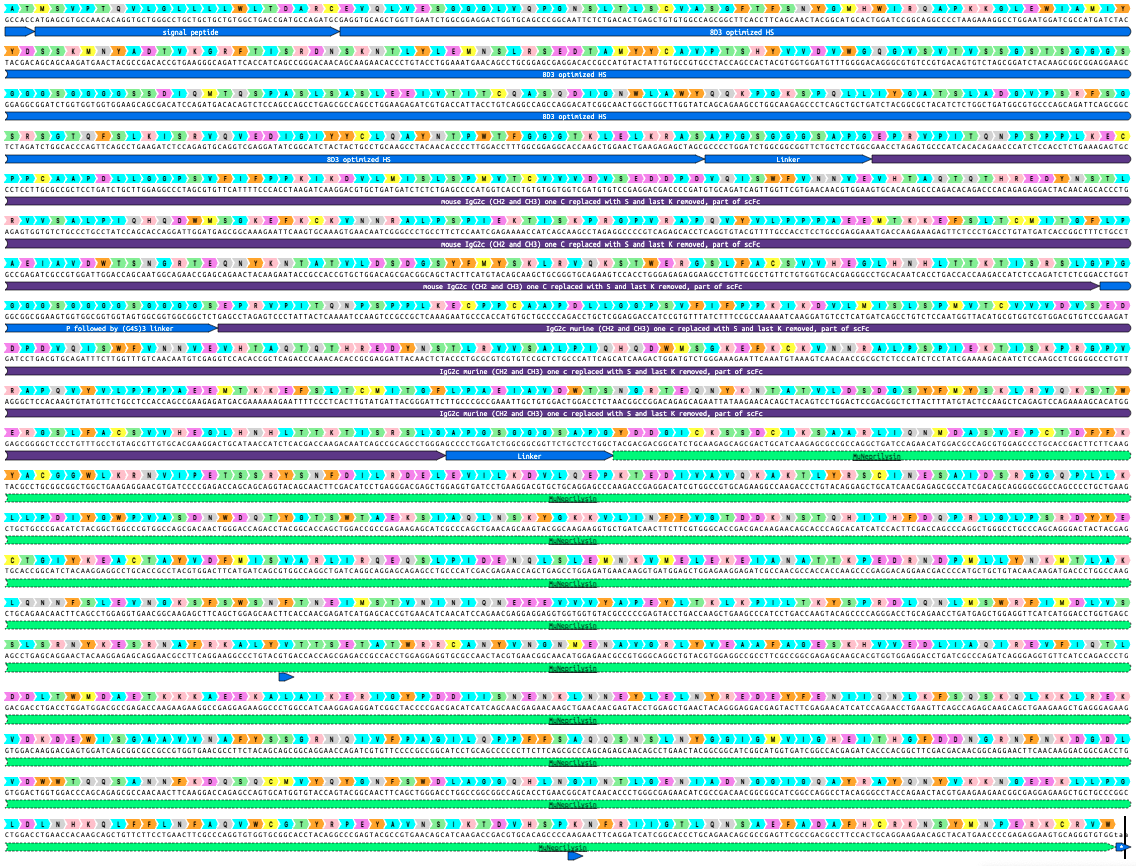
**

B


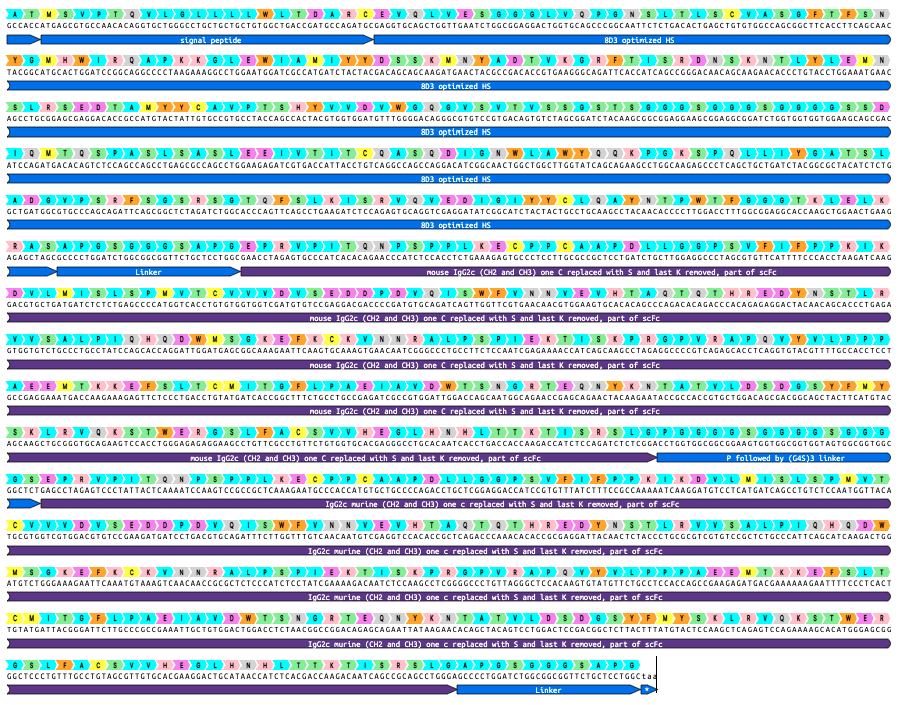


C

**Supplementary Figure 1*.*** The sequences of the genes that was inserted in to the pcDNA3.4 vector used to produce the constructs. The signal peptide is cleaved while expressing the protein so is not left in the final protein that have been used. A. sNEP-scFc-scFv8D3. B. muNEP-scFc-scFv8D3. C. scFc-scFv8D3


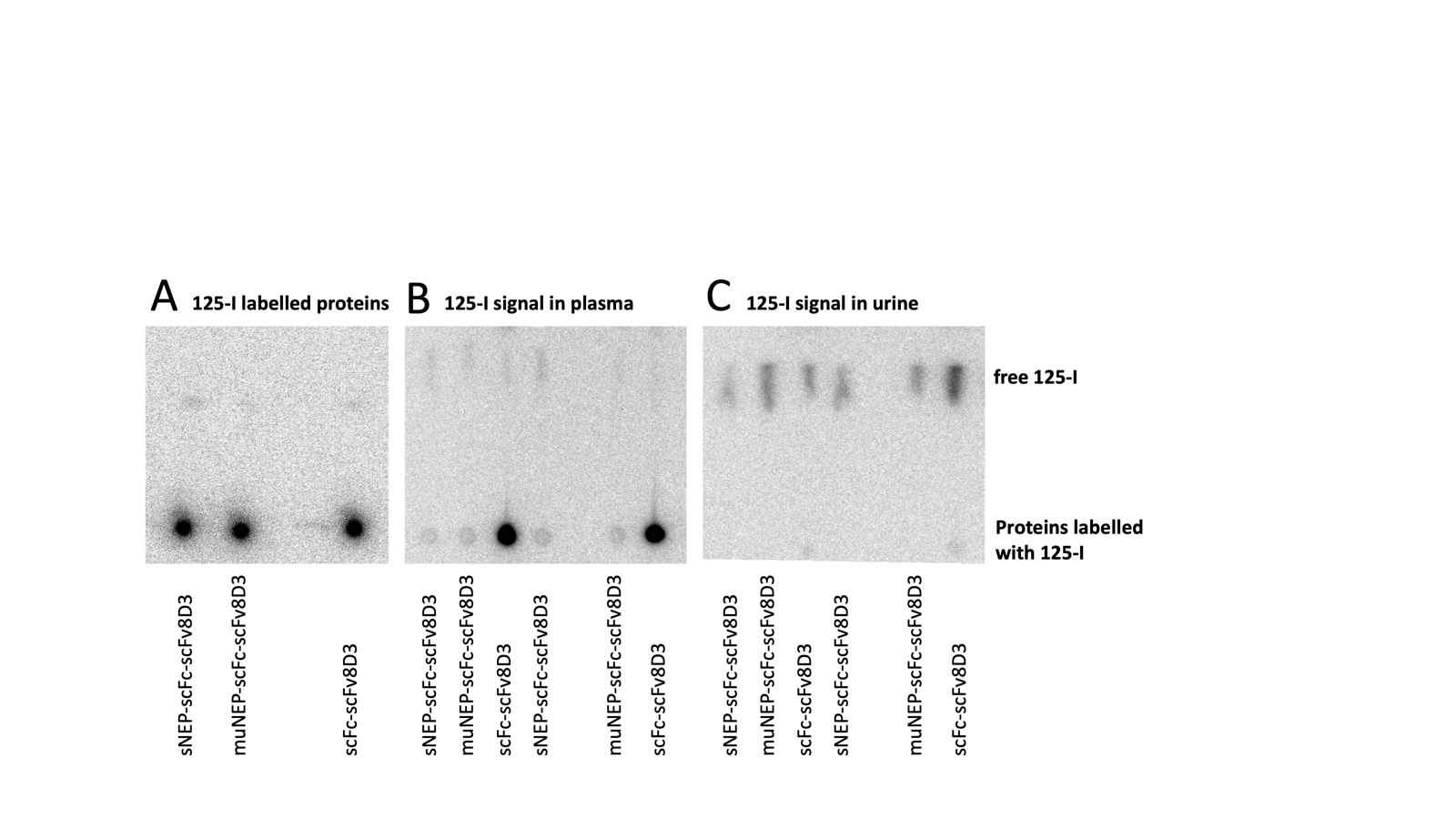


**Supplementary Figure 2.** Instant thin layer chromatography (iTLC) of proteins before and after in vivo experiment. A. Proteins after labelling with ^125^I and before application in vivo study. B. Plasma samples and C. Urine samples 72 hours post injection of 30 nmol/kg body weight of SNEP-scFcscFv8D3, muNEP-scFc-scFv8D3 and scFc-scFv8D, which was applied intravenously in the tail vein. All samples were applied on a silica-coated aluminium plate and separated with 70% (v/v) acetone. The radioactive signal was developed with an X-ray film and red in a Cyclon Phosphoimager.


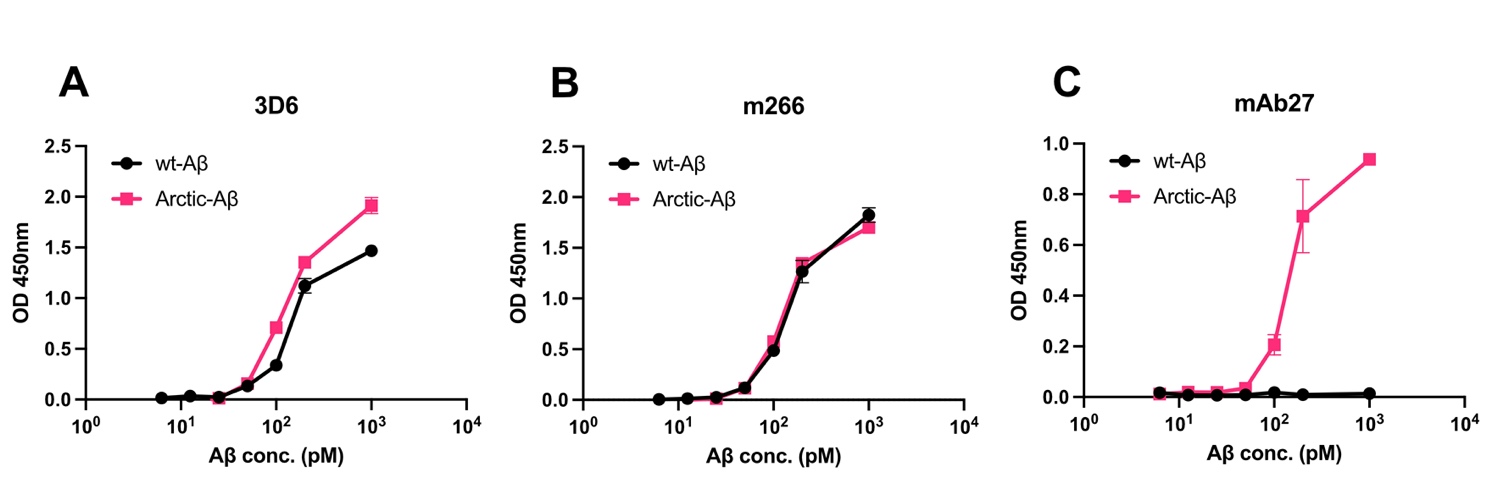


**Supplementary Figure 3.** Binding selectivity of anti-Aβ antibodies used in this study. ELISA plates coated with an anti-Aβ42 capture antibody. Serial dilution of either wild-type Aβ1-42 (wt-Aβ) or arctic-Aβ1-42 added to the plates. **A:** when using 3D6 as the detection antibody, both wt-Aβ and arctic-Aβ could be detected. **B:** when using m266 as the detection antibody, both wt-Aβ and arctic-Aβ could be detected. **C:** when using mAb27 as the detection antibody, only arctic-Aβ could be detected.

**
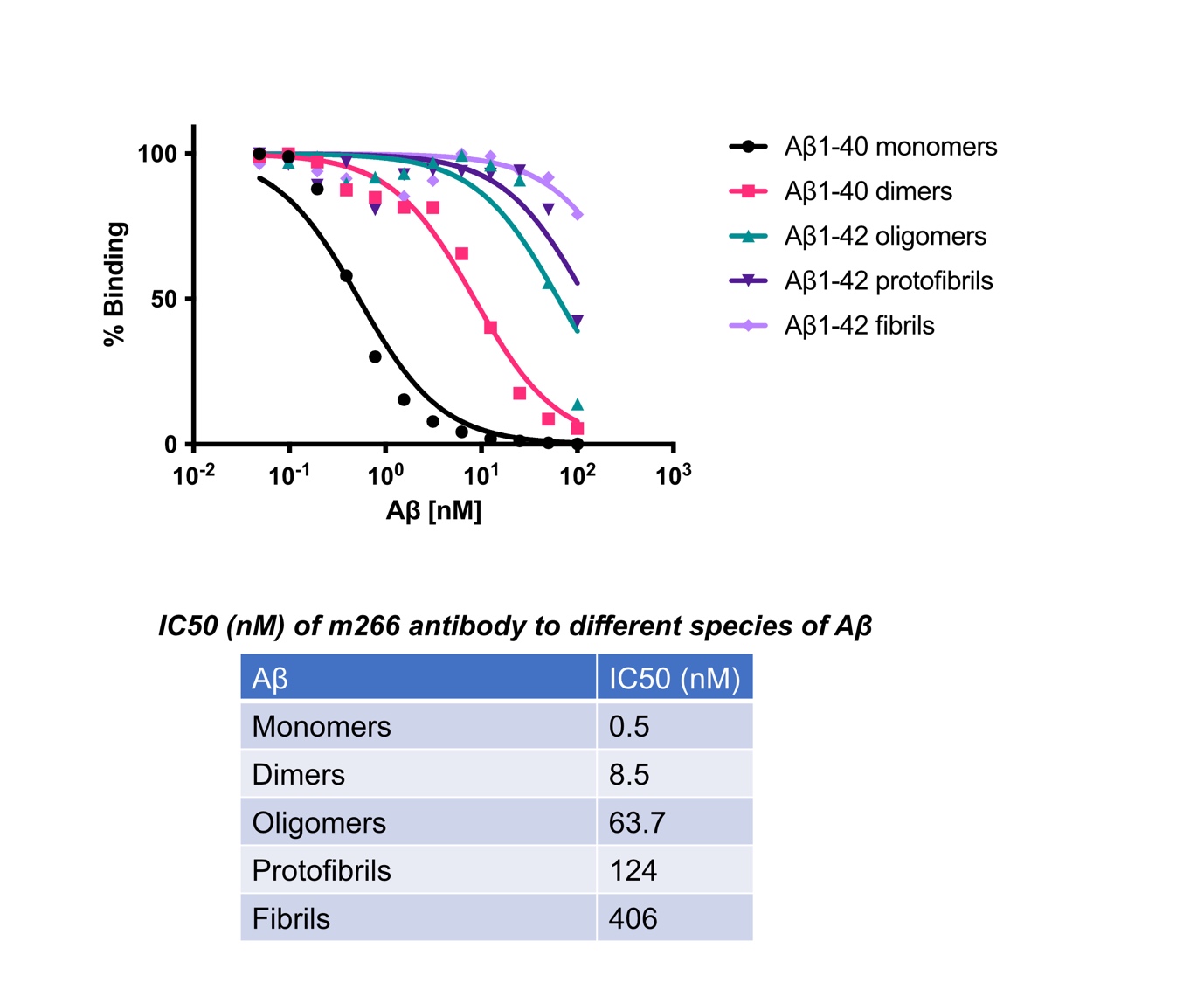
**

**Supplementary Figure 4.** Inhibition ELISA demonstrating the binding strength of m266 antibody to different species of Aβ. Five different Aβ species were used: Aβ1-40 monomers, Aβ1-40 dimers, Aβ1-42 oligomers, Aβ1-42 protofibrils and Aβ1-42 fibrils, prepared as described previously [40]. The assay was performed as described previously [40]. Inhibitory concentration-50 (IC50) of m266 binding to the different Aβ species is present in the table. m266 bound stronger to Aβ monomers compared to other Aβ species. Binding strength of m266 antibody decreased as the size of Aβ species increased.


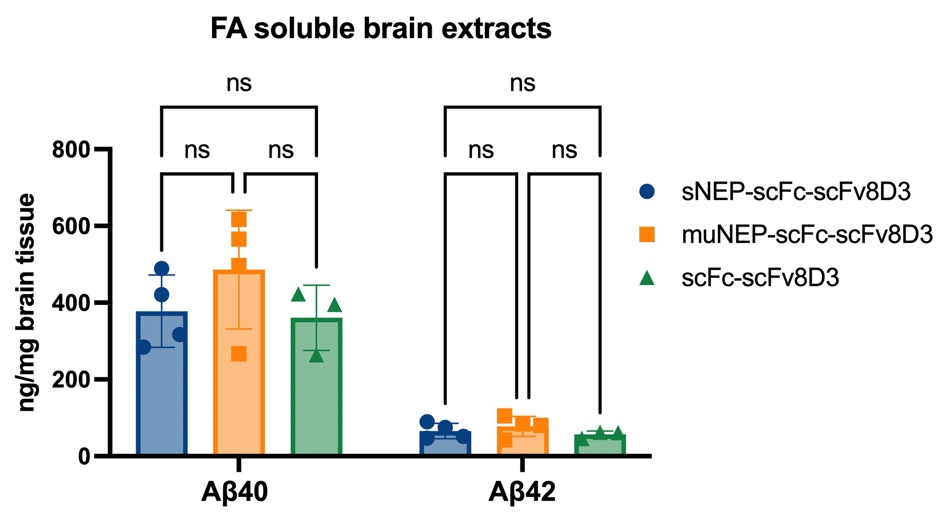


**Supplementary Figure 5.**  Total concentration of Aβ40 and Aβ42 in FA soluble brain extracts of tg-ArcSwe mice following treatment with therapeutic doses of 30 nmol/kg body weight of sNEP-scFc-scFv8D3 or muNEP-scFc-scFv8D3, using scFc-scFv8D3 as the negative control. No significant differences detected among the three groups. Results presented as mean ± SD. One-way ANOVA with Bonferroni’s multiple comparison test was applied (n=4/sNEP-scFc-scFv8D3 and muNEP-scFc-scFv8D3; n=3/scFc-scFv8D3). (p>0.05=ns; p≤0.05= *; p≤0.01= **; p≤0.001= ***).


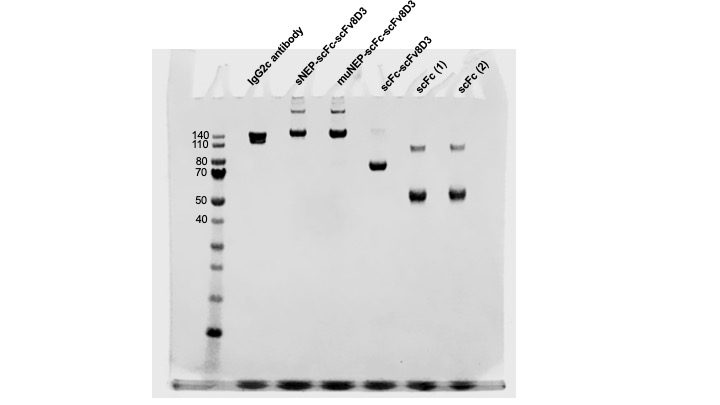


**Supplementary Figure 6.** Complete image of the SDS-PAGE gel presented in Figure 2A.
